# Genome-wide identification and expression of TIFY family in cassava (*Manihot esculenta* Crantz)

**DOI:** 10.3389/fpls.2022.1017840

**Published:** 2022-10-05

**Authors:** Linling Zheng, Qi Wan, Honggang Wang, Changlin Guo, Xiaolei Niu, Xiaofei Zhang, Rui Zhang, Yinhua Chen, Kai Luo

**Affiliations:** ^1^ Hainan Key Laboratory for the Sustainable Utilization of Tropical Bioresources, Hainan University, Haikou, China; ^2^ School of Tropical Crops, Hainan University, Haikou, China; ^3^ CGIAR Research Program on Roots Tubers and Bananas (RTB), International Center for Tropical Agriculture (CIAT), Cali, Colombia

**Keywords:** cassava, TIFY family, sequence analysis, abiotic stress, expression profile

## Abstract

Plant-specific TIFY [TIF(F/Y)XG] proteins serve important roles in the regulation of plant stress responses. This family encodes four subfamilies of proteins, JAZ (JASMONATE ZIM-domain), PPD (PEAPOD), ZML (Zinc-finger Inflorescence-like), and TIFY. In this work, a total of 16 JAZ, 3 PPD, 7 ZML, and 2 TIFY genes were found in cassava (*Manihot esculenta* Crantz) at the genome-wide level. The phylogenetics, exon-intron structure, motif organization, and conserved domains of these genes were analyzed to characterize the members of the JAZ, PPD, and ZML subfamilies. Chromosome location and synteny analyses revealed that 26 JAZ, PPD, and ZML genes were irregularly distributed across 14 of the 18 chromosomes, and 18 gene pairs were implicated in large-scale interchromosomal segmental duplication events. In addition, *JAZ*, *PPD*, and *ZML* gene synteny comparisons between cassava and three other plant species (*Arabidopsis*, *Populus trichocarpa*, and rice) uncovered vital information about their likely evolution. The prediction of protein interaction network and *cis*-acting elements reveal the function of *JAZ*, *PPD*, and *ZML* genes. Subsequently, expression patterns of *JAZ*, *PPD*, and *ZML* genes were validated by qRT-PCR as being expressed in response to osmotic, salt, and cadmium stress. Moreover, almost all JAZ subfamily genes were responsive to jasmonic acid (JA) treatment. In particular, *MeJAZ1*, *MeJAZ13*, and *MeJAZ14*, were highly up-regulated by three treatments, and these genes may deserve further study. This comprehensive study lays the groundwork for future research into TIFY family genes in cassava and may be valuable for genetic improvement of cassava and other related species.

## Introduction

TIFY [TIF(F/Y)XG] gene family is a transcription factor family that is thought to play a key role in a range of biological activities, including plant defense and growth regulation (Shikata et al., 2004; Liu et al., 2020). TIFY family has a highly conserved motif (TIF[F/Y]XG) located in TIFY domain (Vanholme et al., 2007). Previous studies showed that TIFY subfamily, JAZ (JASMONATE ZIM-domain), ZIM/ZML (Zinc-finger Inflorescence Meistem: ZIM and ZIM-like), and PPD (PEAPOD) are the names of four subfamilies within this family based on their domain architecture and overall sequence (Vanholme et al., 2007; Bai et al., 2011). Aside from the TIFY subfamily, which only has the TIFY domain, the other three subfamilies have discrete and conserved domains. The JAZ subfamily comprises a C-terminal conserved domain designated as Jas (SLX2FX2KRX2RX5PY), whose sequence is identical to that of the CCT domain’s N-terminus (Staswick, 2008; Chung et al., 2009). PPD subfamily members have an N-terminal PPD domain, a TIFY domain, and a modified Jas domain missing proline-tyrosine in the C-terminus (Chung et al., 2009). All members of the ZIM/ZML subfamily contain TIFY domains, CCT domains (CONSTANS, CO-like, TOC1), and a C2C2-GATA DNA-BINDING domain (Vanholme et al., 2007; Chico et al., 2008; Staswick, 2008; Chung et al., 2009). To date, TIFY genes have been identified in numerous species, including 18 in *Arabidopsis thaliana* (Vanholme et al., 2007; Chung et al., 2009), 20 in rice (*Oryza sativa*) (Ye et al., 2009), 27 in maize (*Zea mays*) (Bai et al., 2011), 19 in grape (*Vitis vinifera*) (Zhang et al., 2012), 24 in *Populus trichocarpa* (Wang et al., 2017), 49 in wheat (*Tricicum aestivum*) (Ebel et al., 2018), and 36 in *Brassica rapa* (Saha et al., 2016).

Out of four subfamilies, the JAZ subfamily has gained a great deal of attention because of its critical involvement in the jasmonic acid (JA) signaling pathway. When plants respond to developmental or environmental cues, JA-coupled isoleucine (JA-ile) is identified by F-box CORONATINE INSENSITIVE 1 (SCF^COI1^), which subsequently stimulates JAZs degradation by the 26S proteasome, causing the activation of JA-responsive genes for defense to occur (Yan et al., 2013). JAZ proteins inhibit TFs such as MYC2, which enhance JA-responsive gene transcription in plant cells that contain low levels of JA. The NINJA/TPS (new interactor for JAZ/TOPLESS) corepressor complex inhibits downstream genes as a molecular mechanism (Pauwels et al., 2010). *Arabidopsis JAZ* genes are degraded in the SCF^COI1^ complex in response to an accumulation of JA, and the R2R3-MYB transcription factors MYB21 and MYB24 are then released to activate defense genes (Song et al., 2011).

The TIFY gene family is essential for plant growth and development, as well as stress responses. TIFY family genes are important in determining the development of plant organs and tissues such as the stem, leaf, and flower. *AtZML1* and *AtZML2* are transcriptional regulators of developmental events in *Arabidopsis*, and overexpression of *AtZIM* results in longer petioles and hypocotyls (Shikata et al., 2004). *AtPPD1* and *AtPPD2* play critical roles in leaf formation by regulating the arrest of DMC proliferation (White, 2006; Gonzalez et al., 2015; Baekelandt et al., 2018). Overexpression of *JAZ1* or *JAZ4* reduces *Arabidopsis* freezing stress responses by regulating jasmonate signaling pathway (Seo et al., 2011). Rice *OsJAZ1* interacts with and represses *OsbHLH148*, reducing drought tolerance (Seo et al., 2011). *GhJAZ2* interacts with and represses the *GhbHLH171* basic helix-loop-helix (bHLH) transcriptional factors, reducing cotton’s resistance to *Verticillium dahlia* and insect herbivory (He et al., 2018). All of these observations imply that the TIFY gene family plays several regulatory functions in cell signaling and modulating plant responses to stressors, and hence may be a potential resource for stress-responsive genes.

Owing to its edible starch-rich storage root, cassava (2n = 36, *Manihot esculenta* Crantz), a woody Euphorbiaceae shrub, is recognized as the sixth most important food and economic crop in Africa, Asia, Latin America, and the Caribbean (Wilson et al., 2017). Furthermore, because of their high starch content, storage roots are promising candidates for bioethanol production, a critical alternative to fossil fuels (Jansson et al., 2009). Cassava’s extraordinary resistance to a wide range of unfavorable conditions, such as drought and poor fertility soils, making it a source of food security in locations where other food crop species would fail. As a result, for the genetic evolution of stress resistance in cassava and other crops, a deeper understanding of the molecular mechanisms underlying abiotic stress responses in cassava is necessary. TIFY proteins have critical roles in plant growth and stress response. However, there is limited information on the TIFY gene family in cassava. In the present study, genome-wide identification and investigation of the TIFY family genes were carried out from assembled cassava genomes. Phylogenetic, gene structure, conserved domain, chromosomal position, synteny, and *cis*-element research were carried out to provide insight into their evolutionary connections and putative functions. Furthermore, the expression patterns of TIFY genes in different tissues of different cassava varieties were investigated using published transcriptome data, and the expression patterns in cassava in response to osmotic, salt, and cadmium stressors, as well as exogenous JA treatments were investigated using quantitative real-time RT-PCR (qRT-PCR). Our research provides critical information that will aid in the future characterization of TIFY genes in cassava.

## Materials and methods

### Identification and annotation of TIFY family members from cassava

Cassava (*Manihot esculenta* v6.1) genome sequences and annotations were obtained from EnsemblPlants (http://plants.ensembl.org/index.html). TIFY family members in the cassava genome were discovered using two separate methods. First, 18 *Arabidopsis* (Vanholme et al., 2007) and 20 rice TIFY (Ye et al., 2009) amino acid sequences were used as queries in a cassava genomic library BLATP search with an *E*-value of 10^-5^. Second, TIFY domain (PF06200), Jas (PF09425), and CCT motifs (PF06203) HMM profiles were obtained from Pfam (http://pfam.xfam.org/) and used to screen proteins in HMM3.3.1 (http://hmmer.org/, E-value 0.01). (Liu et al., 2020). Thereafter, the two sets of candidates were pooled, redundant proteins were removed, and the Pfam database was used to select candidate proteins based on conserved domains. TIFY proteins’ MW (molecular weight) and *p*I (isoelectric point) were predicted using ExPASy (http://au.expasy.org/tools/pitool.html) (Gasteiger et al., 2003). The secondary structure of cassava TIFY proteins was estimated using the PRABI online tool (http://www.prabi.fr/). The gene ontology (GO) of cassava TIFY genes was analyzed using omicshare online tools (https://www.omicshare.com/tools/Home/Soft/gogsea).

### Multiple sequence alignment and phylogenetic analysis

An unrooted tree was constructed using MEGA7.1 software and the Neighbor-joining technique with 1000 bootstrap replicates at each node utilizing 105 JAZ, PPD, and ZML protein sequences from cassava, *Arabidopsis*, rice, *Populus trichocarpa*, *Brassica napus*, *Gossypium arboretum*, *Vitis vinifera*, and *Brachypodium distachy* (**Supplementary Table 1**). Muscle was used to align the different sequences of cassava JAZ, PPD, and ZML proteins to investigate their sequence-level conservation. Furthermore, all cassava JAZ, PPD, and ZML protein sequences were utilized to build a unique phylogenetic tree for future investigation.

### Conserved motif and gene structure analysis

For the MEME (http://meme.nbcr.net/meme/cgi-bin/meme.cgi) analysis of conserved motifs, the number of conserved motifs was set to 10. InterProScan (http://www.ebi.ac.uk/Tools/pfa/iprscan/) was used to annotate the discovered motifs. An integrated graphic of the phylogenetic tree, conserved motif, and conserved domain was constructed using the TBtools software (Chen et al., 2020). To assess the exon-intron organization of TIFY genes, the *JAZ*, *PPD*, and *ZML* genomic sequences and CDS (coding sequence) acquired from the Phytozome database (https://phytozome-next.jgi.doe.gov) were evaluated in gene structure display server http://gsds.gao-lab.org/ (Hu et al., 2015) programs.

### Chromosomal positions, duplication and synteny analysis

The chromosomal positions of each JAZ, PPD, and ZML member were confirmed using the cassava genome annotation dataset. Tandem duplications and segmental duplications, two processes of gene expansion, were studied to evaluate gene duplication occurrences. Tandem duplications are defined as several genes from the same family that sit in the same or nearby intergenic region. Cassava, *Arabidopsis*, *Populus trichocarpa*, and rice orthologous genes were retrieved. Then, MCScanX analyzed orthologous genes between cassava and other species. The TBtools software estimated the non-synonymous (Ka), synonymous (Ks), and Ka/Ks of each duplicated gene pair and the syntenic TIFY gene pairs; Ka/Ks < 1 indicated purifying selection, Ka/Ks = 1 showed neutral selection, and Ka/Ks > 1 indicated positive selection (Jeffares et al., 2015).

### *Cis*-regulatory element analysis

The TBtools program was used to extract the 2000 bp upstream sequences of the *JAZ*, *PPD*, and *ZML* coding DNA sequences from the cassava genome data, and possible *cis*-acting elements were discovered using the PlantCARE database (http://bioinformatics.psb.ugent.be/webtools/plantcare/index.html) (Lescot et al., 2002).

### Prediction of the cassava TIFY protein-protein interaction network

*Arabidopsis* interologues were used to predict the protein-protein interaction network to examine the relationship of cassava JAZ, PPD, and ZML proteins further. The functional protein association network was analyzed using the STRING online service (https://cn.string-db.org/) with a confidence parameter of 0.15 (Franceschini et al., 2013). Then, the protein-protein interaction was visualized in Cytoscape software (Shannon et al., 2003).

### *In silico* expression analysis *via* RNA-seq data

RNA-seq data from roots and leaves of farmed cultivar Argentina 7 (Arg7), the wild subspecies (W14), and Kasetsart University 50 (KU50), as well as in response to exogenous ABA, were used to evaluate the expression patterns of *JAZ*, *PPD*, and *ZML*. The Sequence Read Archives (SRAs) were collected by the National Center for Biotechnology Information (NCBI) (**Supplementary Table 2** lists the accession numbers). The gene expression levels were determined using FPKM (Fragments Per Kilobase of exon model per Million mapped fragments). Log_2_-transformed heatmaps of all *JAZ*, *PPD*, and *ZML* genes were produced using TBtools (Chen et al., 2020).

### Plant materials and treatments

Cassava South China 205 (SC205), a widely farmed cassava cultivar in China, was used in this study. Three-noded stem segments from 8-month-old cassava plants were subcultured for 40 days on Murashige and Skoog medium at 25°C under a 16 h light/8 h dark cycle at Hainan University (Haikou, Hainan, China). Then, uniform seedlings were subjected to exogenous JA and abiotic stress treatments, respectively. Exogenous JA treatments were conducted by spraying leaves with 50 mM methyl jasmonate (MeJA) for 1 and 6 h post treatment. For abiotic stresses, seedlings were treated with MS medium with 30% polyethylene glycol (PEG) 6000, 400 mM NaCl, and 100 mgL^-1^ CdCl_2_ for 4, 12, and 24 hours (h), respectively, for the osmotic, salt, and cadmium stress treatments. In all cases, untreated seedlings (0 h) acted as controls. Before RNA extraction, the leaf samples were collected, instantaneously frozen in liquid nitrogen, and stored at -80°C.

### RNA extraction and qRT-PCR

The expression patterns of cassava *JAZ*, *PPD*, and *ZML* genes were investigated using qRT-PCR. Total RNAs were extracted from all samples using RNAprep Pure Plant Plus Kit (TIANGEN Biotech Co., Ltd., Beijing) according to the manufacturer’s instructions. Approximately 1 μg of extracted total RNA was used to synthesize cDNA using a reverse transcriptase kit and the manufacturer’s instructions (TIANGEN Biotech Co., Ltd., Beijing). The first strand of cDNA was created using a reverse transcriptase kit (M1631, Thermo, USA). A 7500 Real-Time PCR System with a total reaction volume of 20 μL was used for real-time PCR, which included 2 μL cDNA template, 1 μL forward primer, 1 μL reverse primer, 10 μL qPCR Master Mix, and 6 μL sterilized ddH_2_O. The real-time PCR amplification protocol was set at 95°C for 30 seconds (s) for 40 cycles, 95°C for 5 s, 55°C for 30 s, 72°C for 30 s, and 72°C for 10 minutes (Cao et al., 2022). The ΔΔCT technique described by Schmittgen and Livak (2008) was used to analyze the relative expression levels of *JAZ*, *PPD*, and *ZML* genes, and relative changes in gene expression were computed using *elongation factor 1 alpha* (*EF1α*) as a reference gene. The primers used are listed in **Supplementary Table 3**. Each group was scored on three biological and three technical replications.

### Statistical analysis

The results are expressed as mean ± standard deviation (SD). The Duncan’s multiple range tests were used to analyze the differences between groups. *P*-value ≤ 0.05 is considered to be statistically significant.

## Results

### Identification of TIFY family genes in cassava

BLAST and HMMER searches revealed 28 putative TIFY family genes in the cassava genome (**Table 1**). The Pfam was used to analyze their conserved domains in order to validate these findings and further classify these proteins (**Supplementary Table 4**). While all 28 proteins were discovered to have a TIFY domain, the seven proteins with both a TIFY domain and a CCT motif were also found to have a C2C2-GATA zinc finger (PF00320) and were thus predicted to be members of the ZML subfamily. Three of the 19 TIFY proteins with a Jas motif lacked the conserved PY motif at their C-termini, which is characteristic of a partial Jas domain in PPD proteins (Chung et al., 2009); thus, they were classified as members of the PPD subfamily, while the remaining 16 proteins were classified as members of the JAZ subfamily. The remaining two proteins had a TIFY domain but no Jas or CCT motif, therefore they were classified as members of the TIFY subfamily.

**Table 1:** Details of the TIFY family in cassava.

Gene name	Transcript name	Full CDS length (bp)	Protein			Sublocation(WoLF)
			Protein length (a.a.)	*p*I	Molecular weight (kDa)	
MeJAZ1	Manes.03G042500.1.p	825	275	9.39	29.57	Nucleus
MeJAZ2	Manes.03G055200.1.p	654	218	8.81	23.04	Nucleus
MeJAZ3	Manes.04G147900.1.p	750	250	9.38	26.91	Nucleus
MeJAZ4	Manes.06G004000.1.p	1173	391	9.42	41.31	Nucleus
MeJAZ5	Manes.07G023500.1.p	813	271	9.10	30.13	Nucleus
MeJAZ6	Manes.08G102800.1.p	585	195	8.99	21.82	Chloroplast
MeJAZ7	Manes.09G186200.1.p	606	202	9.76	22.52	Nucleus
MeJAZ8	Manes.11G016700.1.p	795	265	8.75	28.41	Nucleus
MeJAZ9	Manes.14G172400.1.p	1008	336	9.38	35.88	Nucleus
MeJAZ10	Manes.15G122700.1.p	414	138	9.18	15.83	Nucleus
MeJAZ11	Manes.15G122900.1.p	396	132	8.78	14.95	Nucleus
MeJAZ12	Manes.15G133000.1.p	1227	409	9.19	43.62	Nucleus
MeJAZ13	Manes.16G088300.1.p	690	230	8.32	23.82	Mitochondria
MeJAZ14	Manes.16G093500.1.p	831	277	8.96	29.44	Nucleus
MeJAZ15	Manes.17G071600.1.p	459	153	9.57	17.36	Nucleus
MeJAZ16	Manes.17G082100.1.p	1128	376	8.51	39.87	Nucleus
MePPD1	Manes.03G173500.1.p	1023	341	7.59	37.39	Nucleus
MePPD2	Manes.05G081400.1.p	1026	342	7.65	38.19	Nucleus
MePPD3	Manes.15G032500.1.p	822	274	7.7	29.6	Nucleus
MeTIFY1	Manes.03G065500.1.p	1266	422	9.74	44.88	Chloroplast
MeTIFY2	Manes.16G067800.1.p	1311	437	9.15	45.47	Nucleus
MeZML1	Manes.05G189500.1.p	1068	356	4.79	38.97	Nucleus
MeZML2	Manes.05G189600.1.p	858	286	6.45	31.04	Nucleus
MeZML3	Manes.07G041200.1.p	1095	365	4.73	40.16	Nucleus
MeZML4	Manes.07G041300.1.p	891	297	5.24	31.4	Nucleus
MeZML5	Manes.10G097400.1.p	915	305	5.28	32.21	Nucleus
MeZML6	Manes.18G056300.1.p	846	282	6.89	30.47	Nucleus
MeZML7	Manes.18G056400.1.p	924	308	5.63	33.56	Chloroplast
MeTIFY1	Manes.03G065500.1.p	1266	422	9.74	44.88	Chloroplast
MeTIFY2	Manes.16G067800.1.p	1311	437	9.15	45.47	Nucleus

Overall, we found 16 JAZ, 3 PPD, 7 ZML, and 2 TIFY proteins in cassava (**Table 1**; **Supplementary Table 5**). These genes were designated for their chromosomal positions: *MeJAZ1* through *MeJAZ16*, *MePPD1* through *MePPD3*, *MeZML1* through *MeZML7*, and *MeTIFY1* through *MeTIFY2*. The lengths of the CDS ranged from 396 bp (MeJAZ11) to 1311bp (MeTIFY2). Similarly, the amino acid sequence lengths range from 132 a.a. (MeJAZ11) to 437 a.a. (MeTIFY2), and the expected molecular weights range from 14.95 kDa (MeJAZ11) to 45.47 kDa (MeTIFY2). The fact that the anticipated *p*I of 21 of 28 cassava TIFYs was more than 7.0 suggests that the vast majority of cassava TIFYs are alkaline. Based on subcellular localization data, the majority of cassava TIFY proteins (23 of 28) were anticipated to be found in the nucleus. Tao et al. (2022) reported that, except for one ZML protein predicted to be located in the mitochondrion, all other kiwifruit (*Actinidia eriantha* and *A. chinensis*) TIFYs were predicted to be located in the nucleus, which is similar with the present study. The secondary structure analysis discovered that random coil was the most common secondary structure in cassava TIFY amino acid sequences, accounting for up to 73.77% (MeJAZ12), followed by alpha helix, extended strand, and beta turns. The secondary structure of the cassava TIFY protein was made up of 64% random coil, 21% alpha helix, 11% extended strand, and 4% beta twists, respectively (**Supplementary Table 6**). Because the study was primarily focused on TIFY family members with either CCT or Jas motifs, proteins anticipated to belong to TIFY subfamilies (MeTIFY1 and MeTIFY2) with just a TIFY domain were not investigated further.

### Phylogenetic analyses of the JAZ, PPD, and ZML members from eight species

**Figure 1:**
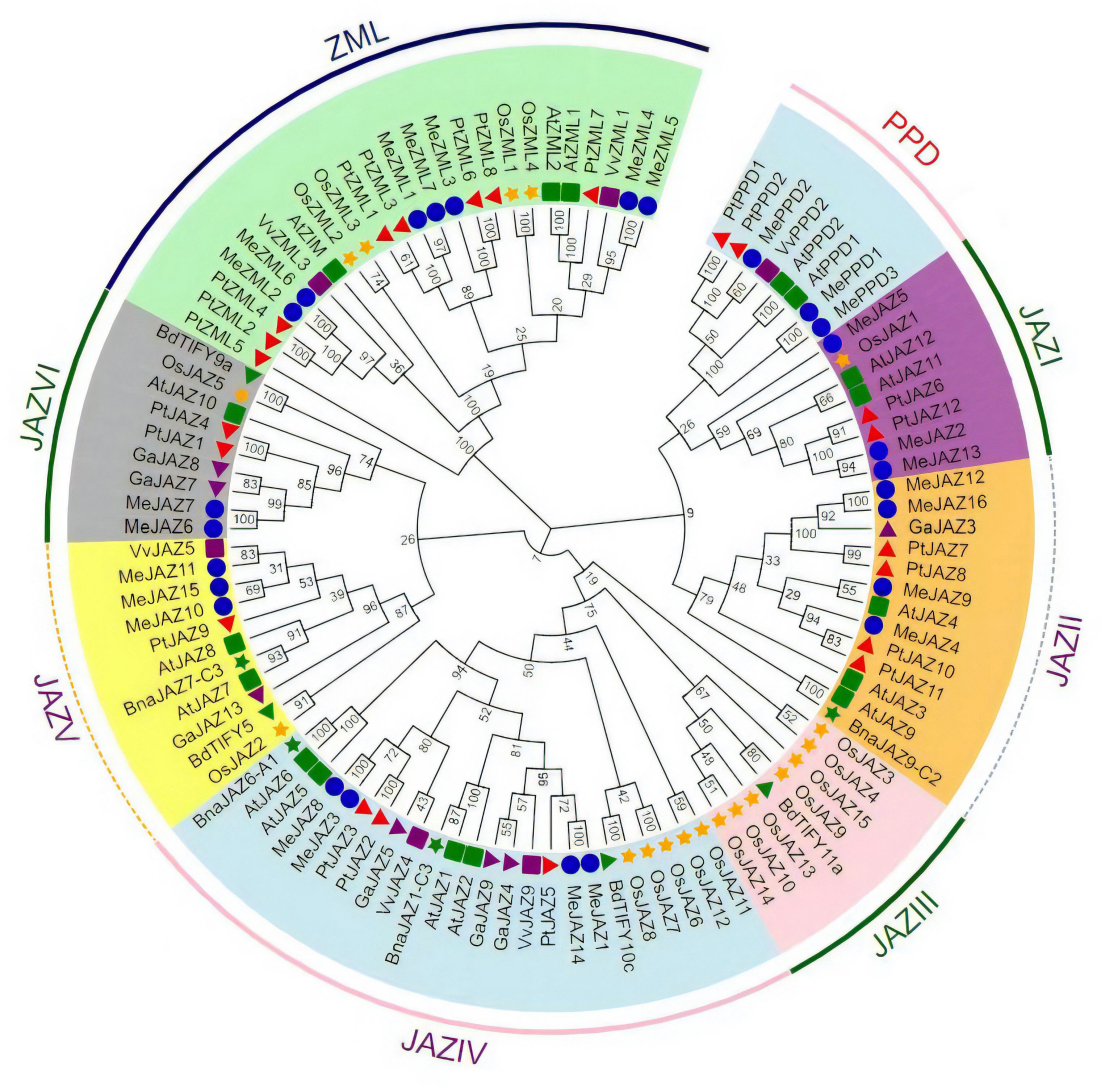
Phylogenetic tree of the TIFY proteins predicted in cassava and those previously identified in *Arabidopsis*, rice, *Populus trichocarpa*, *Gossypium arboretum, Vitis vinifera, Brachypodium distachyon*, and *Brassica napus*. The phylogenetic tree was constructed using the Neighbor-joining (NJ) method with 1000 bootstrap replications as implemented in MEGA7.1 from a TIFY protein sequence alignment. Different clade was distinguished by different color. Me, *Manihot esculanta*; At, *Arabidopsis thaliana*; Os, *Oryza sativa*; Pt, *Populus trichocarpa*; Ga, *Gossypium arboretum*; Vv, *Vitis vinifera*; Bd, *Brachypodium distachyon*; Bna, *Brassica napus*.

To assess the evolutionary relationship of TIFY genes between cassava and other plant species, a neighbor-joining phylogenetic tree was generated using multiple sequence alignment of TIFY protein sequences from eight different plant species, including 26 in cassava, 17 in *Arabidopsis*, 19 in rice, 22 in *Populus trichocarpa*, four in *Brassica napus*, seven in *Gossypium arboretum*, six in *Vitis vinifera*, and four in *Brachypodium distachy* (**Supplementary Table 1**). Based on their evolutionary links, the 105 proteins were divided into three primary clades: JAZ, PPD, and ZML. JAZ proteins were the largest group, with 73 JAZ proteins divided into six clades: I, II, III, IV, V, and VI; ZIM and ZML proteins formed one clade, while PPD proteins formed a distinct clade (**Figure 1**). JAZI and JAZV each had three MeJAZs, JAZII and JAZIV each had four MeJAZs, JAZVI had two MeJAZs. Out of the six clades in the JAZ subfamily, JAZ III clade contains JAZ proteins from two monocotyledon taxa (**Figure 1**).

### Sequence analysis of cassava JAZ, PPD, and ZML members

**Figure 2:**
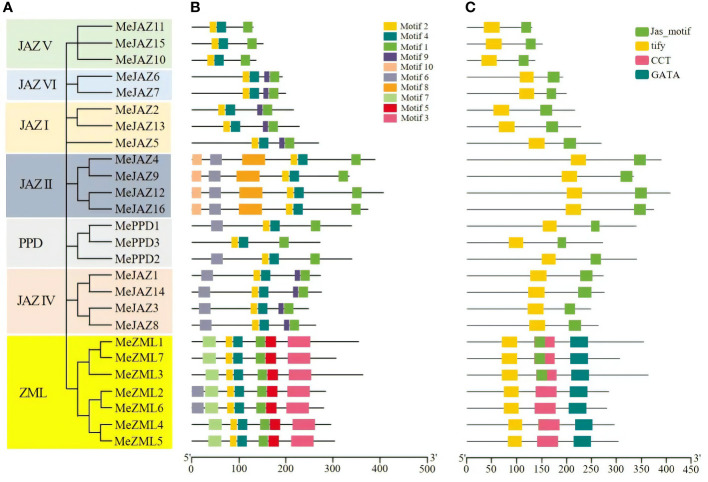
Phylogenetic relationships, conserved motifs and conserved domains of the predicted cassava JAZ, PPD, and ZML proteins. **(A)** The phylogenetic tree of cassava JAZ, PPD, and ZML proteins constructed with the Neighbor-joining (NJ) method in MEGA 7.1. The bootstrap values were 1,000 replications for major branches. The genes in seven subgroups were marked with different colors. **(B)** Different motif compositions of cassava JAZ, PPD, and ZML proteins were detected using MEME. The boxes with different colors on the right denote 10 motifs. A detailed motif introduction is shown in **Supplementary Table S7**. **(C)** Conserved domains in the cassava ZML, PPD and JAZ proteins. Different domains were shown in different color.

Further phylogenetic analysis was carried out utilizing just genomes from JAZ, PPD, and ZML members in cassava (**Figure 2A**). The results revealed that the tree’s structure was comparable to the phylogenetic tree created using JAZ, PPD, and ZML sequences from the eight plant species shown in **Figure 1**. We illustrated the distribution of their exons, motifs, and conserved domains to support the evolutionary relationships between the cassava JAZ, PPD, and ZML proteins.

The exon-intron structure of all found *JAZ*, *PPD*, and *ZML* genes was studied to better understand the structural diversity of the TIFY gene family in cassava. Members of the cassava TIFY gene family vary in terms of the number of exons and the length of introns, as illustrated in **Supplementary Figure 1**. The number of exons varied between one and ten. The second intron of *MePPD3* was the longest of any gene sequence, resulting in a genomic DNA sequence of 13,228 kb. The gene exon-intron organization also demonstrated a significant relationship between phylogeny and exon-intron structure, such that genes with comparable exon-intron structures belonged to the same phylogenetic group. For example, the genes in the subgroups JAZI, JAZII, and JAZVI have 5, 7, and 6 exons, respectively (**Supplementary Figure 1**).

Using the MEME suite, ten conserved motifs in cassava JAZ, PPD, and ZML proteins were identified and tagged based on their *E*-values (**Figure 2B**). The details of 10 motifs are provided in **Supplementary Table 7**. The number of preserved motifs varies from 3 to 7 among JAZ, PPD, and ZML members. All members of the cassava JAZ, PPD, and ZML proteins shared motif 1, motif 2, and motif 4. Furthermore, certain motifs were restricted to specific groups, such as motif 3, motif 5, and motif 7, which were restricted to the ZML clade, and motif 8 and motif 10, which were restricted to the JAZII subgroup (**Figure 2B**). Motif 1 was predicted to have a CCT structure, whereas motifs 2 and 4 were predicted to have a TIFY structure and motif 3 was predicted to have a GATA zinc-finger structure (**Supplementary Table 7**). Individuals within the same group or subgroup had the same type and number of motifs, and their distribution was typically the same (**Figure 2B**).

Furthermore, the conserved domains in cassava JAZ, PPD, and ZML proteins were evaluated using the Pfam, and four potential conserved domains were identified: the tify domain, the Jas motif domain (also known as CCT 2), the CCT domain, and the GATA domain (**Figure 2C**). These four potential conserved domains were found in multiple sequences and showed group/subgroup selectivity. For example, the majority of ZML clade members have at least three conservative domains, including the TIFY domain, CCT domain, and GATA domain. The JAZ subfamily was predominantly composed of the TIFY domain and the Jas motif domain, whereas the PPD subfamily was mostly composed of the TIFY and Jas domains (**Figure 2C**). The core sequences of the TIFY domain differ across members of the cassava TIFY family. Our findings show that all members of the JAZ, PPD, as well as TIFY subfamilies uncovered in this study have the distinctive “TIFYXG” pattern (**Supplementary Figure 2**). According to our findings, the TIFY domain of proteins from the JAZ, PPD and TIFY subfamilies contains the consensus sequence TIFYXG, whereas the majority of cassava ZML proteins have the conserved pattern TLS[F/Y]XG. Furthermore, multiple sequence alignment identified two variations of the TIFY domain in ZML subfamilies, “TLTFXG” and “TIAFXG” (**Supplementary Figure 2**) (Bai et al., 2011). In general, the classification of these proteins based on domain composition accorded well with the previous phylogenetic investigation.

### Chromosomal locations and duplication events of cassava *JAZ*, *PPD*, and *ZML* genes

**Figure 3:**
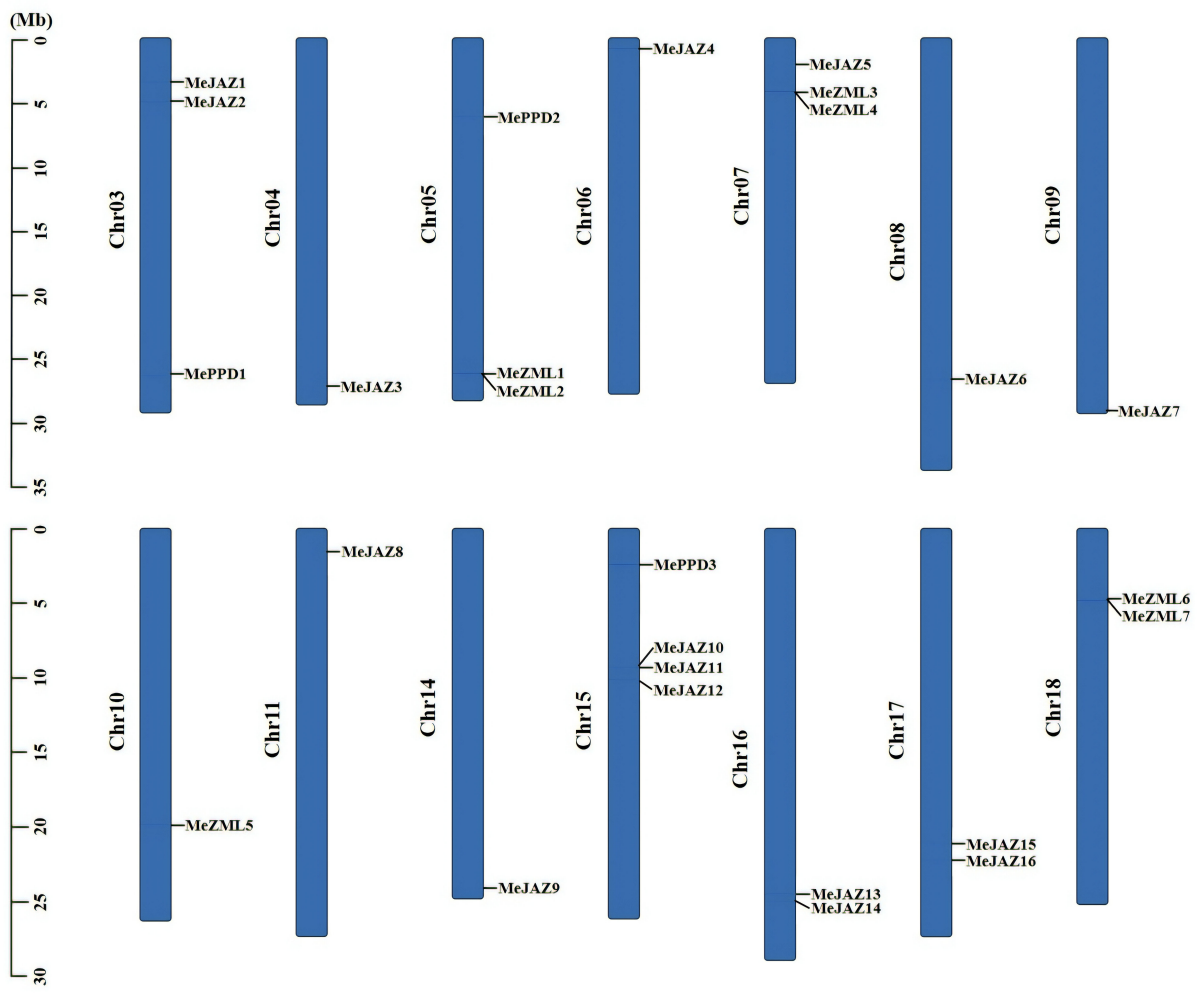
Distribution of *ZML*, *PPD* and *JAZ* genes on cassava chromosomes. The chromosome numbers were shown at the left of each chromosome. The genes were listed on the left of the chromosomes. The scale on the left is in million bases (Mb).

**Figure 4:**
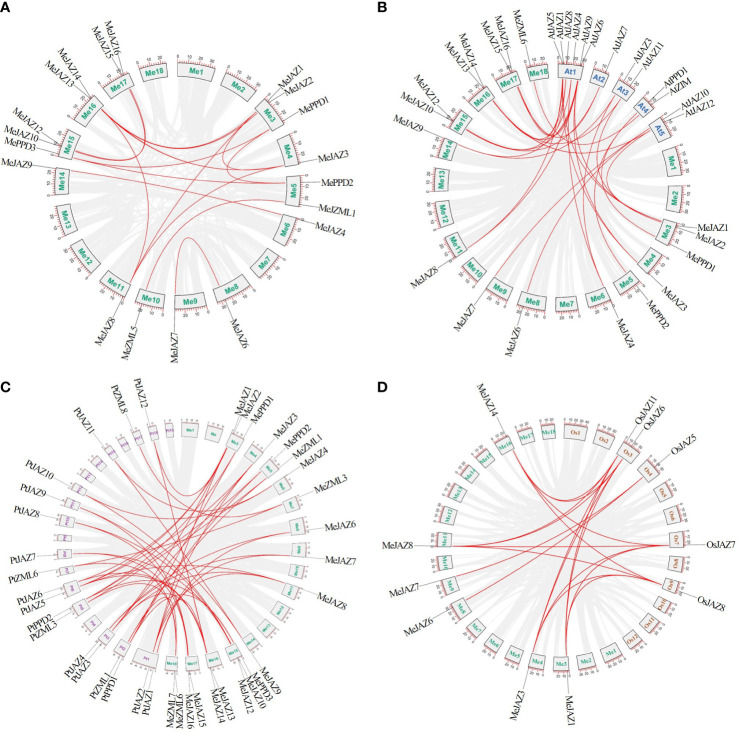
Synteny analysis of *ZML*, *PPD* and *JAZ* genes. **(A)** Orthologous relationships between cassava and its genome ancestors. **(B)** The syntenic *ZML*, *PPD* and *JAZ* genes between cassava and *Arabidopsis*. **(C)** The syntenic *ZML*, *PPD* and *JAZ* genes between cassava and *Populus trichocarpa*. **(D)** The syntenic *ZML*, *PPD* and *JAZ* genes between cassava and rice. The cassava, *Arabidopsis*, *Populus trichocarpa*, and rice chromosomes are shown in different color boxes and labeled Me (green), At (blue), Pt (purple), and Os (orange), respectively. The gray line in the background indicates a collinear block in the genome of cassava and other plants, while the red line highlights the isomorphic gene pair.

To characterize the genomic distribution of the cassava *JAZ*, *PPD*, and *ZML* genes, the cassava plant genome’s pseudochromosome assembly was used to map their chromosomal locations to the duplicated blocks. The 26 *JAZ*, *PPD*, and *ZML* genes were unequally distributed across 14 of cassava’s 18 chromosomes (**Figure 3**). Chr15 contains the most distributed genes, with four members, followed by Chr03, Chr05, and Chr07, all of which have three members. Chr16, Chr17, and Chr18 each have two members, whereas the remaining seven chromosomes (Chr04, Chr06, Chr08, Chr09, Chr10, Chr11, and Chr14) each have only one (**Figure 3**). Due to the significant genetic variety within this family, genetic recombination and exchanges play critical roles in the propagation of TIFY genes. Gene duplications have an impact on the expansion of gene families. A single gene can be replicated twice, according to our findings. We counted two pairings if a gene has two duplicated occurrences in distinct places. An examination of intra-species gene duplication revealed that the cassava *JAZ*, *PPD*, and *ZML* genes had 18 pairs of duplicate genes (**Figure 4**; **Supplementary Table 8**). We found no tandem duplication clusters, indicating that tandem duplication is unlikely to be a significant contributor to the expansion of the *JAZ*, *PPD*, and *ZML* genes in cassava. Furthermore, Ka/Ks ratios were calculated to assess the possible selection pressure exerted on the discovered duplicated gene pairs. All of the Ka/Ks ratios of the previously stated segmentally duplicated cassava *JAZ*, *PPD*, and *ZML* gene pairs were less than one (**Supplementary Table 8**), demonstrating that cassava’s repetitive TIFY family genes were substantially constrained by significant purifying selection pressure.

### Synteny and evolutionary analyses of cassava and other plants *JAZ*, *PPD*, and *ZML* genes

A set of comparative syntenic graphs of cassava connected with two dicots plants (*Arabidopsis* and *Populus trichocarpa*) and a monocot plant (rice) were produced to study the possible evolutionary implications of the cassava TIFY gene family. When cassava was compared to other species, a substantial number of syntenic blocks were discovered. There were 21 and 17 syntenic linkages between cassava TIFY genes and those of *Populus trichocarpa* and *Arabidopsis*, followed by rice, which has 6 genes (**Figure 4**; **Supplementary Table 9**). There were 46, 28, and 18 cassava orthologous gene pairs in *Populus trichocarpa*, *Arabidopsis*, and rice, respectively. Despite the fact that there were more syntenic gene pairs between cassava and dicots than between cassava and monocots, six collinear genes (*MeJAZ1*, *MeJAZ3*, *MeJAZ6*, *MeJAZ7*, *MeJAZ8*, and *MeJAZ14*) were found in both dicots and monocots (**Figure 4**; **Supplementary Table 9**).

### Analysis of *cis*-elements in cassava *JAZ*, *PPD*, and *ZML* genes

Transcriptional regulation of gene expression by *cis*-elements in the promoter region may be critical in plant response to environmental stresses (Walther et al., 2007). Furthermore, phytohormones such as salicylic acid (SA), ethylene (ET), abscisic acid (ABA), and JA are required for plant response to abiotic stresses (Santner and Estelle, 2009). In this study, we projected probable *cis*-elements of 26 cassava *JAZ*, *PPD*, and *ZML* genes in promoter regions (**Supplementary Figure 3**; **Supplementary Table 10**). In the promoter regions, nine stress-responsive (MYB, MYC, TC-rich repeats, DRE core, MBS, STRE, W box, WUN-motif, and LTR) and ten hormone-responsive (ABRE, CGTCA-motif, TGACG-motif, GARE-motif, P-box, TATC-box, ERE, TCA-element, TGA-element and AuxRR-core) *cis*-acting elements were identified (**Supplementary Figure 3**; **Supplementary Table 10**). The number of *cis*-elements in the promoter regions of cassava *JAZ*, *PPD*, and *ZML* genes varied. In JAZ clades, for example, the *MeJAZ4* promoter includes 12 different types of *cis*-elements, but the *MeJAZ6* promoter only has four. Overall, the promoter regions of all of these genes contain at least two types of environmental stress responsiveness and one type of phytohormone-related *cis*-elements, implying that these genes may be involved in responses to various abiotic stressors; however, this needs to be confirmed through experimental studies (**Supplementary Figure 3**; **Supplementary Table 10**).

### Protein-protein interaction network of JAZ, PPD, and ZML proteins in cassava

**Figure 5:**
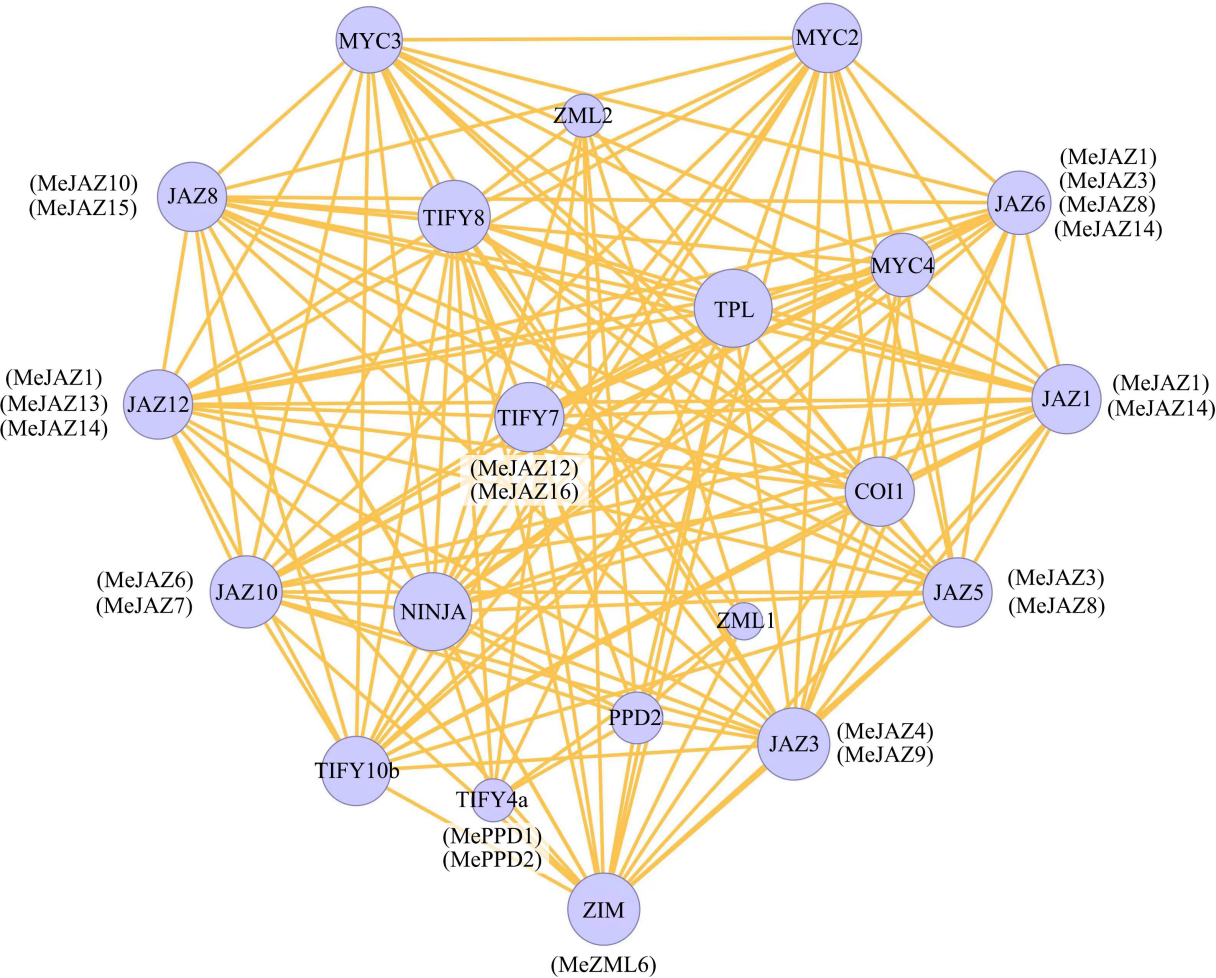
Protein–protein interaction network of cassava JAZ, PPD, and ZML proteins based on their orthologs in Arabidopsis. The color scales represent the relative signal intensity scores. Predicted functional partners exhibited in the network, including NINJA (NOVEL INTERACTOR OF JAZ), TPL (TOPLESS), COI1 (CORONATINE INSENSITIVE 1), and MYC (myelocytomatosis).

STRING was used to build an interaction network to understand the interaction of cassava JAZ, PPD, and ZML proteins on the basis of the orthologues in *Arabidopsis*. The orthologous STRING proteins with the highest bit score were identified using all JAZ, PPD, and ZML protein sequences. According to statistical analysis of 26 cassava JAZ, PPD, and ZML proteins, the homologous proteins match the highest bit score by default, which identified 10 (JAZ1, JAZ3, JAZ5, JAZ6, JAZ8, JAZ10, JAZ12, TIFY4a, TIFY7, and ZIM) proteins involved in the TIFY family networks in *Arabidopsis* (**Figure 5**). Among the 10 detected homologous proteins in *Arabidopsis*, JAZ3 had more interaction partners, including members of the TIFY gene family and other genes, such as MYC, COI1 and NINJA. The findings may shed light on the functions of unidentified proteins.

### GO functional analysis of cassava *JAZ*, *PPD*, and *ZML* genes

OmicShare was utilized to perform GO functional analysis on all cassava *JAZ*, *PPD*, and *ZML* genes for functional annotation. The terms “biological processes”, “cellular components”, and “molecular functions” refer to gene or gene product qualities that help us understand the numerous molecular actions of proteins (Ashburner et al., 2000). According to our findings, the 26 *JAZ*, *PPD*, and *ZML* genes were assigned 24 GO categories (**Supplementary Figure 4**; **Supplementary Table 11**). The most common groups under the “biological process” category were “metabolic process” (23, 88.5%), “regulation of biological process” (19, 73.1%), “biological regulation” (19, 73.1%), and “cellular process” (17, 65.4%). The most common category in the category of cellular components was “cell” (9, 34.6%), followed by “cell part” (8, 30.8%) and “organelle” (8, 30.8%). In the realm of molecular function, 22 (84.6%) genes were categorized as “binding,” with “nucleic acid binding transcription factor activity” (2, 7.7%), and “catalytic activity” (1, 3.8%). (**Supplementary Figure 4**; **Supplementary Table 11**). According to the findings, the cassava TIFY family genes have a range of roles in cellular metabolism.

### Expression profiling of cassava *JAZ*, *PPD*, and *ZML* genes in different tissues

**Figure 6:**
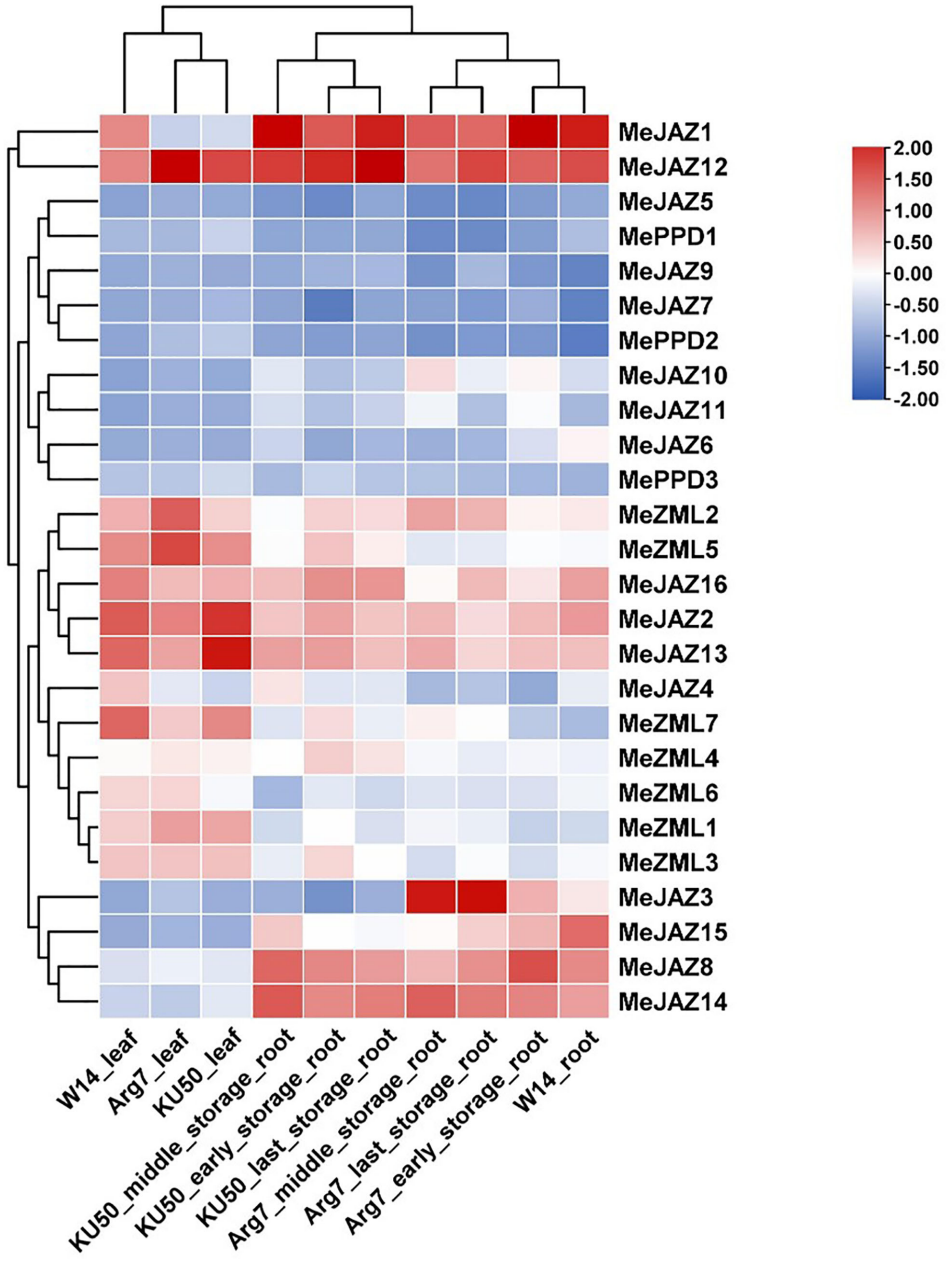
Expression profiles of cassava ZML, PPD and JAZ genes in leaves and roots of Argentina 7 (Arg7), wild subspecies (W14), and Kasetsart University 50 (KU50). Log2 transformed FPKM value was used to create the heat map. The scale represents the relative signal intensity of FPKM values.

The transcript levels of cassava *JAZ*, *PPD*, and *ZML* genes in leaf (3) and root (7) tissues of the farmed cultivar Argentina 7 (Arg7), the wild subspecies (W14), and Kasetsart University 50 (KU50) were investigated using ten publicly available transcriptomes (**Supplementary Table 2**) (Wang et al., 2014). In general, cassava *JAZ*, *PPD*, and *ZML* gene expression revealed a tissue pattern with relatively low expression levels in leaf tissues and increased expression levels in root tissues (**Figure 6**; **Supplementary Table 12**). TIFY family genes with high expression levels are thought to perform critical activities. Most of the cassava *JAZ*, *PPD*, and *ZML* genes were expressed more strongly in Arg7 leaf tissue than in KU50 and W14. In root tissue, the majority of cassava *JAZ*, *PPD*, and *ZML* genes were expressed at higher levels in W14 than in Arg7 and KU50. *MeJAZ2*, *MeJAZ12*, *MeJAZ13*, and *MeZML5* exhibited high expression levels (FPKM value > 3) in leaf tissue in Arg7, W14, and KU50; and *MeJAZ1*, *MeJAZ2*, *MeJAZ8*, *MeJAZ12*, *MeJAZ13*, *MeJAZ14*, *MeJAZ15*, and *MeJAZ16* had high expression levels (FPKM value > 3) in early storage root tissue in Arg7 and KU50. These genes showed constitutive expression for a given tissue in both wild subspecies and domesticated cultivars, indicating that they may play important roles in tissue development or function. Notably, *MeJAZ12* showed relatively high transcript abundance (FPKM value > 3) in all studied tissues in Arg7, W14, and KU50, indicating that the gene may be involved in plant growth and development (**Figure 6**; **Supplementary Table 12**).

### Expression profiling of cassava *JAZ*, *PPD*, and *ZML* genes in response to exogenous ABA treatments

RNA-seq data from cassava leaves subjected to exogenous ABA, which is known to play an important role in plant responses to many types of abiotic stress, were examined. The expression profile of cassava *JAZ*, *PPD*, and *ZML* genes with and without exogenous ABA treatment determined by FPKM values (**Supplementary Figure 5**: **Supplementary Table 5**). Exogenous ABA treatment altered the expression levels of cassava *JAZ*, *PPD*, and *ZML* genes to various degrees. *MeJAZ1*, *MeJAZ2*, *MeJAZ12*, *MeJAZ13*, *MeJAZ14*, *MeJAZ16*, and *MeZML7* all displayed comparable expression patterns and were active with or without exogenous ABA treatment. With the exception of *MeJAZ2* and *MeJAZ16*, the expression of these genes increased following exogenous ABA treatment, suggesting that they may be controlled at an ABA-independent transcriptional level and so perform comparable or distinct roles.

### Expression pattern of cassava JAZ subfamily genes in response to JA treatment

**Figure 7:**
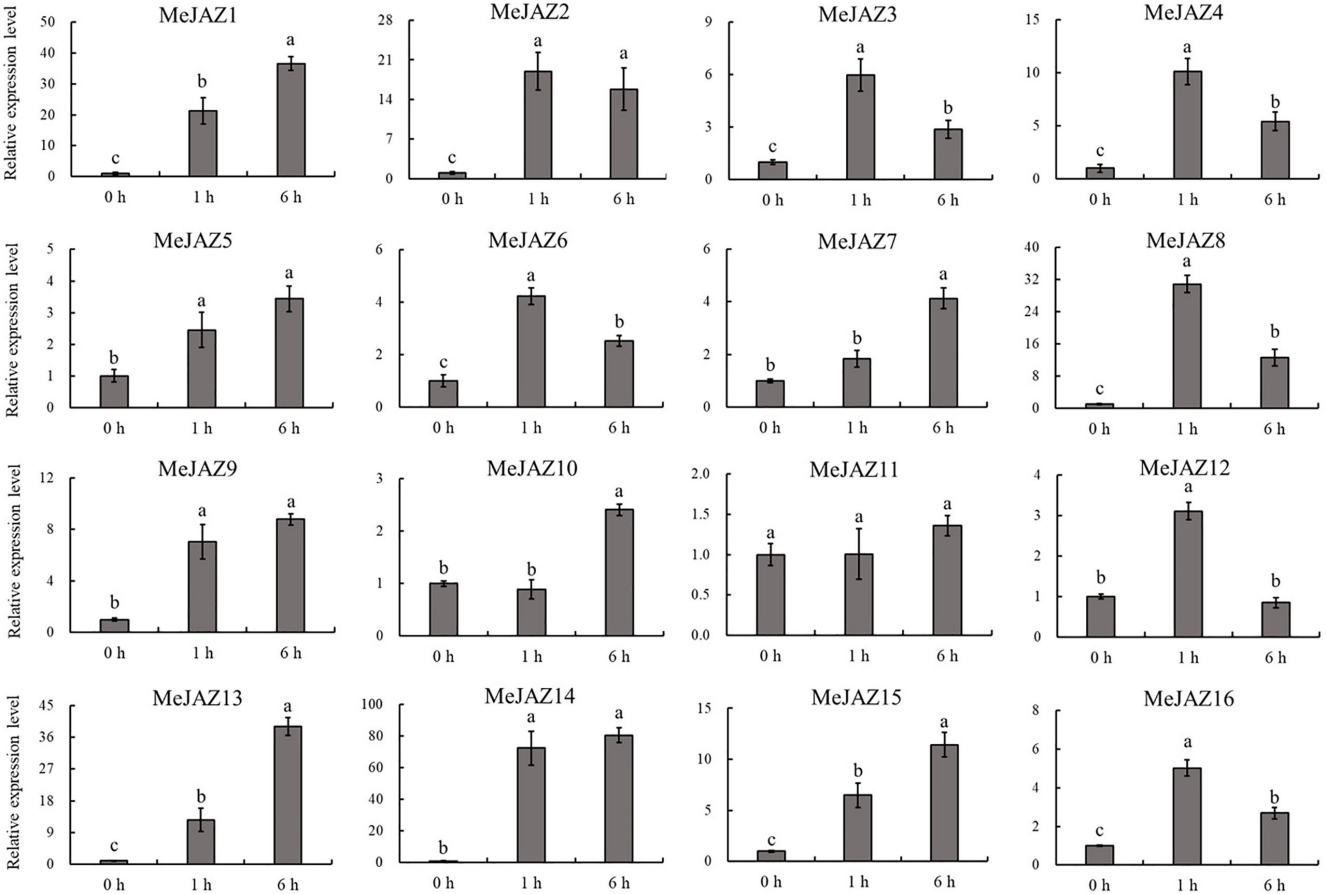
Expression profiles of 16 JAZ genes under exogenous JA treatment in cassava as determined by qRT-PCR. The error bars represent the standard error of the means of three independent replicates. Values denoted by the same letter did not differ significantly at P < 0.05 according to Duncan's multiple range tests.

The JAZ subfamily is an important regulatory factor that controls the initiation of JA by responding to JA stimulation. To explore the response of JAZ subfamily genes identified from cassava with JA application, the transcript level changes of 16 *MeJAZ* gene were investigated by qRT-PCR with the leaves treated with MeJA. Our results showed that almost all JAZ subfamily genes were induced by JA application, with the exception of *MeJAZ11* (**Figure 7**). Among these *MeJAZ* genes, *MeJAZ1*, *MeJAZ2*, *MeJAZ3*, *MeJAZ4*, *MeJAZ5*, *MeJAZ6*, *MeJAZ7*, *MeJAZ8*, *MeJAZ9*, *MeJAZ13*, *MeJAZ14*, *MeJAZ15*, and *MeJAZ16* exhibited an elevated expression pattern at all time points. However, certain members, such as *MeJAZ2*, *MeJAZ3*, *MeJAZ4*, *MeJAZ6*, *MeJAZ8*, *MeJAZ12*, and *MeJAZ16*, showed a similar dynamic which gradually increasing within 1 h, followed by a considerable decrease in expression level than the peak time point. Notably, *MeJAZ14* displayed the most prominent changes in expression levels in response to JA treatment.

### Expression pattern of cassava *JAZ*, *PPD*, and *ZML* genes under abiotic stresses

TIFY family genes have critical roles in plant responses to abiotic stresses, according to new research (Saha et al., 2016). To identify the abiotic-responsive TIFY genes in the cassava genome, RNA was isolated from the leaves of cassava seedlings treated with osmotic, salt, and cadmium, and the relative expression patterns of 26 cassava *JAZ*, *PPD*, and *ZML* genes were evaluated using qRT-PCR.

**Figure 8:**
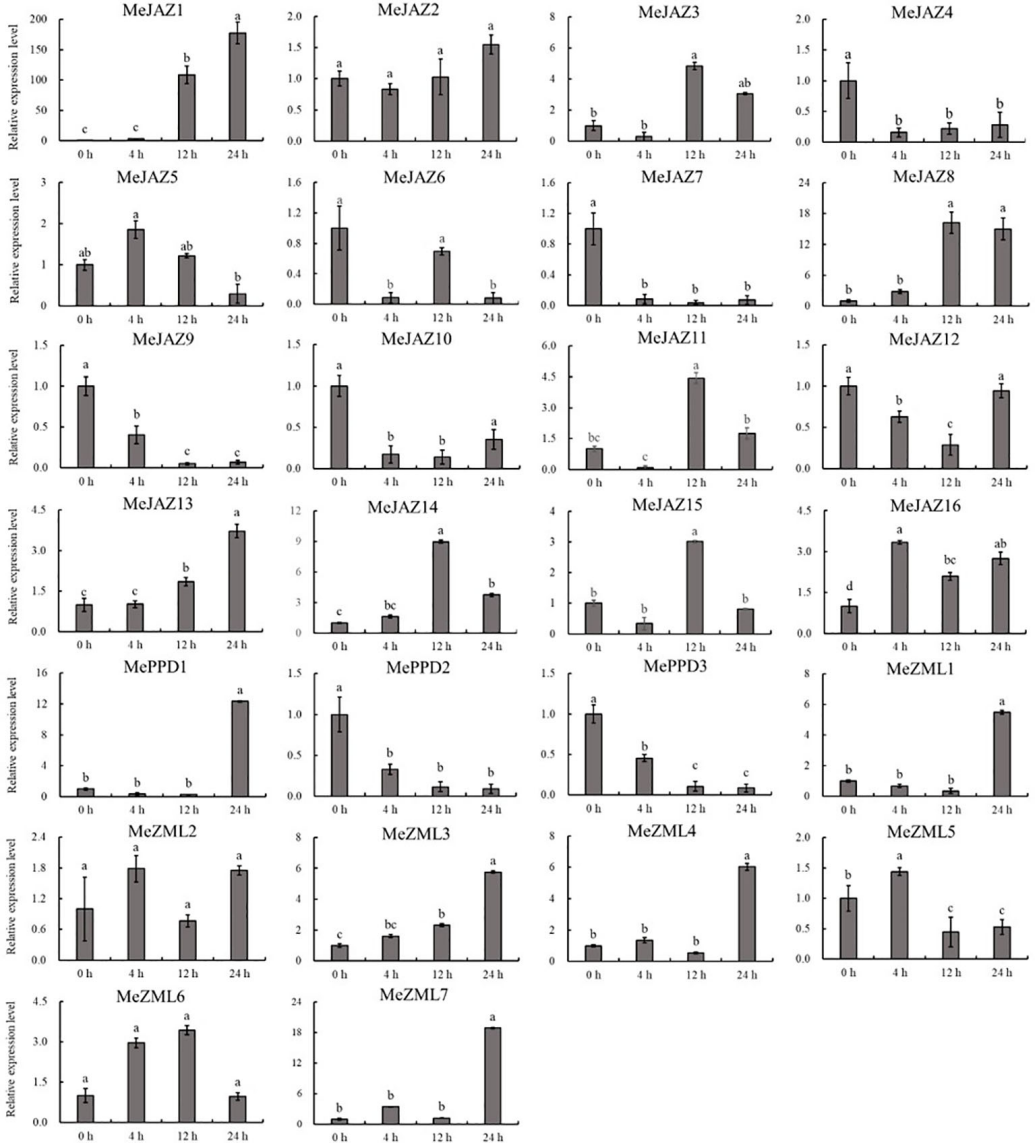
Expression profiles of 26 *ZML*, *PPD* and *JAZ* genes in response to osmotic stress in cassava as determined by qRT-PCR. The error bars represent the standard error of the means of three independent replicates. Values denoted by the same letter did not differ significantly at *P* < 0.05 according to Duncan’s multiple range tests.

**Figure 9:**
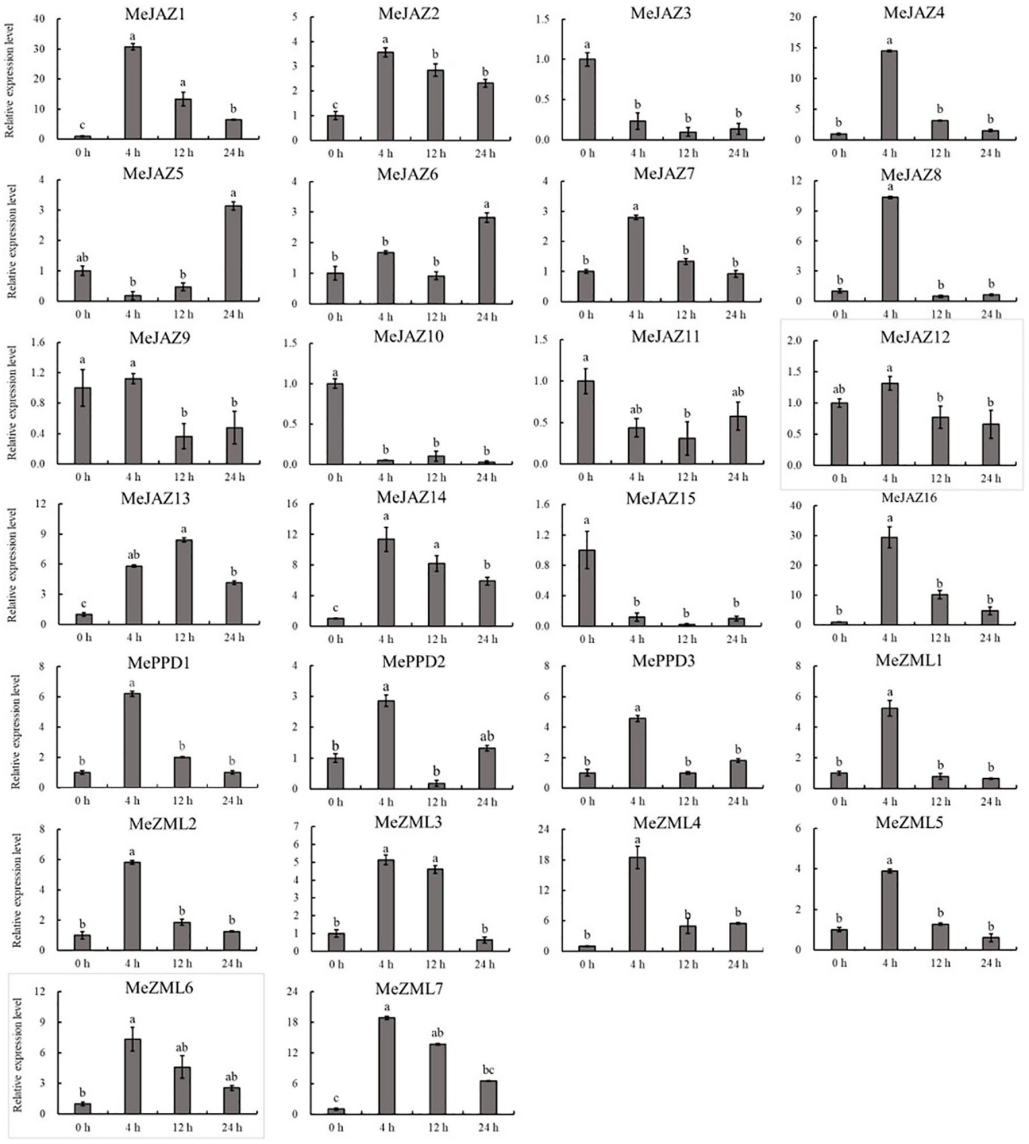
Expression profiles of 26 *ZML*, *PPD* and *JAZ* genes in response to salt stress in cassava as determined by qRT-PCR. The error bars represent the standard error of the means of three independent replicates. Values denoted by the same letter did not differ significantly at *P* < 0.05 according to Duncan’s multiple range tests.

**Figure 10:**
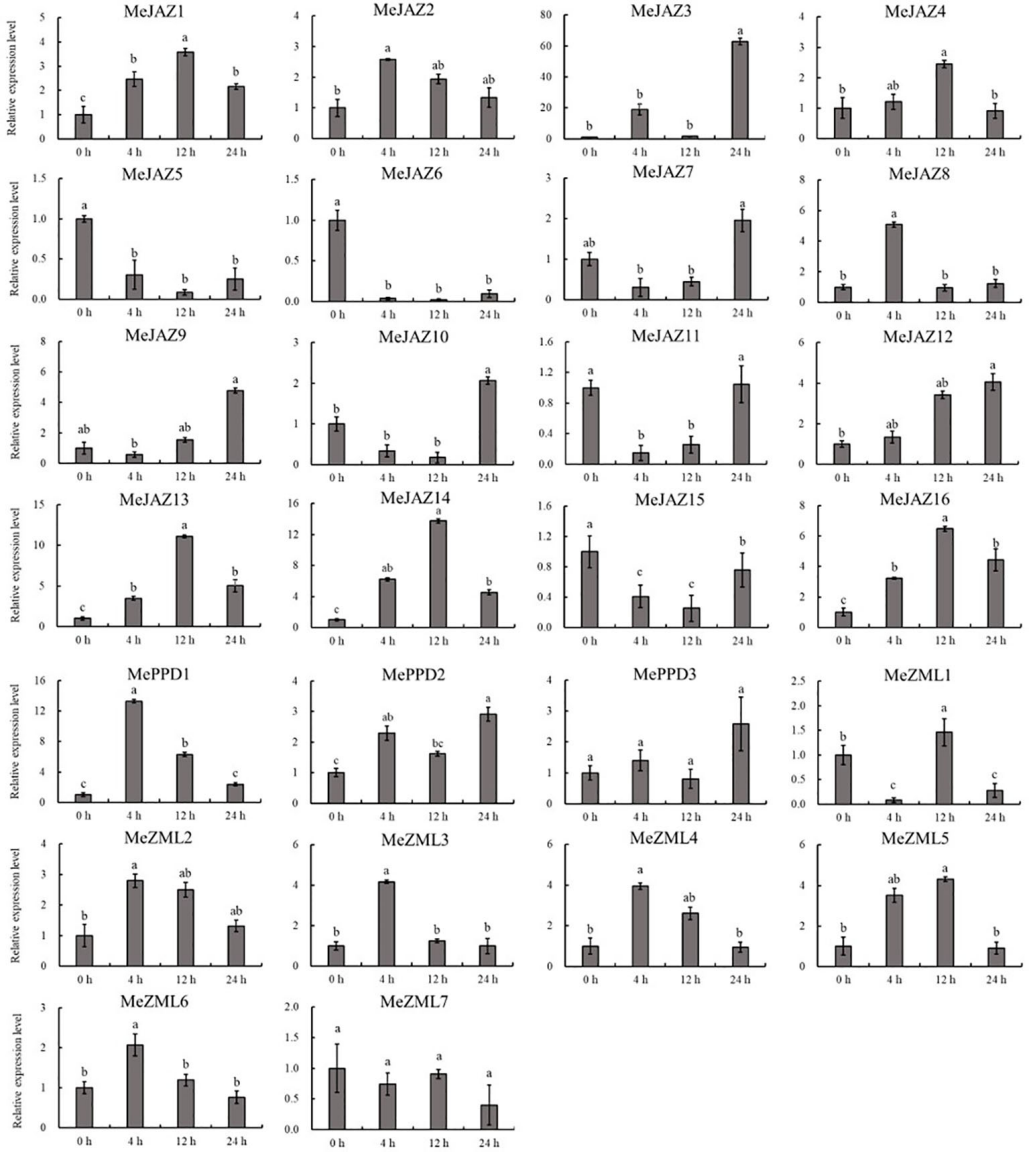
Expression profiles of 26 *ZML*, *PPD* and *JAZ* genes in response to cadmium stress in cassava as determined by qRT-PCR. The error bars represent the standard error of the means of three independent replicates. Values denoted by the same letter did not differ significantly at *P* < 0.05 according to Duncan’s multiple range tests.

The relative expression levels of 10 of 26 genes increased overall in response to osmotic treatment (**Figure 8**). Three of them (*MeJAZ1*, *MeJAZ13*, and *MeZML3*) exhibited an elevated expression pattern, with *MeJAZ1* having the greatest expression. The expression levels of *MeJAZ4*, *MeJAZ6*, *MeJAZ7*, *MeJAZ9*, *MeJAZ10*, *MeJAZ12*, *MePPD2* and *MePPD3* were much lower than the control. Interestingly, ten of the twenty-six genes (*MeJAZ3*, *MeJAZ5*, *MeJAZ8*, *MeJAZ11*, *MeJAZ14*, *MeJAZ15*, *MeJAZ16*, *MeZML2*, *MeZML5*, and *MeZML6*) tended to trend higher and peaked 4 and 12 hours following osmotic stress treatment (**Figure 8**). In comparison to osmotic stress, the cassava JAZ, PPD, and ZML gene families were expressed at greater abundance under salt stress treatment (**Figure 9**). *MeJAZ1*, *MeJAZ2*, *MeJAZ4*, *MeJAZ7*, *MeJAZ8*, *MeJAZ9*, *MeJAZ12*, *MeJAZ14*, *MeJAZ16*, *MeZML1*, *MeZML2*, *MeZML3*, *MeZML4*, *MeZML5*, *MeZML6*, *MeZML7*, *MePPD1*, *MePPD2*, and *MePPD3* exhibited enhanced expression and early up-regulation after 4 h, followed by reduced expression after 12 or 24 h. *MeJAZ5* expression was lowered after 4 and 12 h, but promptly increased after 24 h under salt stress. *MeJAZ1* and *MeJAZ16* showed relative greater expression in response to salt stress than other genes, with 30-fold higher expression than the control (**Figure 9**). Despite the fact that TIFY family genes are substantially implicated in environmental stress tolerance, few reports on TIFY family genes deal with heavy metal tolerance. The majority of cassava TIFY genes, including *MeJAZ1*, *MeJAZ2*, *MeJAZ4*, *MeJAZ8*, *MeJAZ13*, *MeJAZ14*, *MeJAZ16*, *MeZML2*, *MeZML3*, *MeZML4*, MeZML5, *MeZML6*, and *MePPD1*, showed a similar dynamic under cadmium treatment, gradually increasing within 4 h or 12 h and then decreasing with a significantly lower expression level than the peak time point (**Figure 10**). However, *MeJAZ3*, *MePPD2* and *MePPD3* exhibited early up-regulation after 4 h, followed by decreasing expression after 12 h and peaking at 24 h. After cadmium treatment, *MeJAZ12* demonstrated a continuous upregulation trend that peaked at 24 h (**Figure 10**). Interestingly, some cassava TIFY genes, including *MeJAZ1*, *MeJAZ13*, and *MeJAZ14*, were consistently up-regulated at all time points under all three stress situations, indicating that these genes may be involved in the regulation of certain common signaling pathways for diverse stress responses.

## Discussion

The TIFY gene family, being a plant-specific transcription factor family, regulates plant development, physiological processes, and stress response, with the JAZ subfamily being the most well-studied to date (Yan et al., 2009; Ye et al., 2009; Demianski et al., 2012). A better understanding of the TIFY family at the molecular level might aid in deciphering the processes behind stress resistance and developing novel plant varieties with increased resilience. Nonetheless, little is known about the expression and function of this gene family in cassava, the world’s sixth most important staple crop and a tropical crop with a considerable economic effect (Wang et al., 2014; Wilson et al., 2017). As a result, we intended to perform a genome-wide search for TIFY genes in cassava and gain insight into their evolutionary history and expression diversity in a variety of stress-related circumstances.

At least 28 TIFY genes were found in the cassava genome using a BLAST search and HMMER analysis. The number of members in a gene family can be determined by the genome’s size and ploidy level. For example, wheat, a hexaploid plant, has 49 TIFY genes, and *Gossypium hirsutum*, a diploid plant, has 50 TIFY genes (Zhao et al., 2016; Ebel et al., 2018). The found TIFY proteins are highly diverse in terms of their physiochemical properties and domain distribution in cassava, which was consistent with previous studies. TIFY family members have shown high protein sequence diversity in *Arabidopsis* (Vanholme et al., 2007), rice (Ye et al., 2009), and kiwifruit (Tao et al., 2022), indicating that these genes diverged early in land plant evolution and have subsequently suffered changes that have affected their activities. According to subcellular localization studies, the majority of cassava TIFY family members are located in the nucleus, showing that the TIFY gene family developed in plant cells to manage a variety of tasks (Bai et al., 2011; Cai et al., 2020). Remarkably, the TIFY family is found only in terrestrial plants, not green algae or non-photosynthetic eukaryotes (Vanholme et al., 2007; Bai et al., 2011). This shows that plant TIFY homologs evolved after aquatic plants adapted to terrestrial life. Previous study has shown that proteins from different transcription factor families share certain areas (Riechmann et al., 2000). Furthermore, the TIFY TFs may be divided into four subfamilies based on their different domain and motif designs: JAZ, PPD, ZML, and TIFY; however, not all subfamilies are found in all species. The TIFY subfamily, for example, is absent in *Brachpodium distachyon* and *Sorghum bicolor*, whereas the PPD subfamily is found exclusively in dicotyledons and the spikemoss *Selaginella moellendorffii* and not in monocotyledons (Ye et al., 2009). PPD proteins have a role in *Arabidopsis* cell cycle and cell proliferation regulation (White, 2006). Several other genes in monocots are most likely compensating for the molecular activities done by *PPD* genes (Bai et al., 2011). Cassava, like other dicot plants, such as *Populus trichocarpa*, walnut (*Juglans regia*), and peach (*Prunus persica*), has members of all four subfamilies based on domain traits (Wang et al., 2017; Liu et al., 2022; Sheng et al., 2022).

Phylogenetic study indicated that the JAZ subfamily, as well as the ZML and PPD subfamily, all belong to the same lineage. This classification, together with the findings of a study of conserved domains, suggests that their functions have evolved through time. Furthermore, the JAZ subfamily of cassava proteins was classified into six distinct subgroups (**Figure 1**; **Figure 2A**), which is consistent with the findings of a previous study in which JAZ proteins from some species, including apple (*Malus* × *domestica*) (Li et al., 2015), *Populus trichocarpa* (Wang et al., 2017), and kiwifruit (Tao et al., 2022). Previous research has shown that the exon-intron structure may be used to provide further support for phylogenetic groupings, as this type of divergence typically plays a substantial part in the establishment of gene families (Shiu and Bleecker, 2003). According to the phylogenetic tree, the majority of cassava TIFYs within the same clade have a similar pattern of exon-intron structure, even though the number of exons and length of specific introns varies. This occurrence suggested that the intron structure of homologous genes was more likely to be retained during evolution. *MeJAZ1* and *MeJAZ14*, *MeJAZ6* and *MeJAZ7*, and *MeZML4* and *MeZML5* were, for example, clustered together and comprised the same number of exons with almost identical exon lengths. We did, however, find intron/exon loss/gain in several TIFY gene clades. For example, *MeZML1* and *MeZML3* contain 10 exons compared to 7 or 8 exons in all other *MeZML* members, indicating that this subfamily may have gained at least two extra exons throughout evolution (**Supplementary Figure 1**).

Gene duplication helps to the expansion of gene families (Wang et al., 2013). Previous studies found that gene duplications within the TIFY family differed significantly between species. For example, watermelon, *Populus trichocarpa*, and *Brassica rapa* have 4, 10, and 18 duplicated TIFY genes, respectively (Saha et al., 2016; Wang et al., 2017; Yang et al., 2019). A total of 18 duplicated gene pairs were detected in cassava *JAZ*, *PPD*, and *ZML* genes in this study, indicating that gene duplication may be the key mechanism for the formation of the TIFY family in cassava (**Figure 4A**). Only segmental duplication events were detected in the cassava TIFY gene family, as in other species such as *Brassica rapa* (Saha et al., 2016), supporting the concept that duplication events play a substantial role in the development and extension of the TIFY gene family in plants. Furthermore, the Ka/Ks ratios of the duplicated paralogous gene pairs indicated that all duplicated pairs in the cassava TIFY gene family had been influenced by considerable purifying selection, which might have resulted in preserved functionality or pseudogenization. All duplicated blocks in cassava were collinear, indicating that these duplication events may have originated from segmental or large-scale duplication/duplication events, according to intraspecies synteny studies (Wang et al., 2018). Although the duplicated cassava TIFY family genes identified in this study have a common ancestor, we cannot conclude that they will have the same functions and expression patterns because duplicated genes, if they survive, tend to diverge in both their regulatory and coding regions during evolution, resulting in paralogues with different functions (Xu et al., 2012). Several paralogous couples displayed similar expression patterns, despite some variances. The expression pattern of pair of paralogous gene of *Brachypodium distachyon*, *BdTIFY11e* and *BdTIFY11f*, increased gradually with the increase of time under drought treatment (Zhang et al., 2015). Our study found that one pairs of homologous gene (*MeJZA1* and *MeJAZ14*) shown parallel expression patterns under salt and cadmium treatment (**Figure 9**; **Figure 10**). Interestingly, the two pairs of homologous gene contained different *cis*-acting elements. However, *MeZML1* and *MePPD1*, and *MeZML4* and *MeZML7* were not paralogous gene pairs, but sharing the same expression pattern, which was consistent with findings for *Brassica napus* (He et al., 2020).

The transcriptional regulation of genes, which may be influenced by *cis*-elements in the promoter region, governs the responses to diverse stimuli (Ahmadizadeh and Heidari, 2014; Heidari et al., 2019). The presence of multiple *cis*-regulatory elements in the promoter region of cassava TIFY genes demonstrated that these genes may be activated in response to a variety of stressors and several plant hormone response mechanisms, as well as play a role in cassava growth and development. Protein-protein interactions showed that TIFY family members in cassava have significant relationships with TF MYCs. The functions of the JAZ proteins are connected to jasmonate responses, which block jasmonate signals by interacting with the transcription factors MYC2 and MYC3, which regulate the expression of downstream genes (Yan et al., 2007; Melotto et al., 2008; Bai et al., 2011). Furthermore, MYC2 is linked to transcriptional reprogramming and stress tolerance (Verma et al., 2020; Gautam et al., 2021). MYC2 and MYC3 were in the center of the interaction network, interacting with JAZ3, JAZ8, and JAZ12. In this study, MeJAZ4 and MeJAZ9 were syntenic paralogs of JAZ3 in cassava; MeJAZ10 and MeJAZ15 were syntenic paralogs of JAZ8; and MeJAZ1, MeJAZ13, and MeJAZ14 were syntenic paralogs of JAZ12 (**Figure 5**). These cassava JAZs may interact with MYC, suggesting a major contribution to the control of many biological processes. Several TIFY family proteins, especially members from JAZ subfamily, interaction with the critical coronatine-insensitive COI1, a subunit of Skp1/Cullin/F-box SCF^COI1^ E3 ubiquitin ligase, regulates ubiquitin-dependent protein catabolic and fatty acid biosynthetic processes, both of which are essential for protein function and plant reproduction and viability (Heidari et al., 2021). These proteins are critical for protein-protein interactions with COI1, which creates the COI1-JA-Ile-JAZ co-receptor complex with the JA-Ile (jasmonoyl-L-isoleucine) ligand and then forms the COI1-JAZ co-receptor complex with the COI1-JA-Ile-JAZ co-receptor complex (Chini et al., 2007; Thines et al., 2007; Yan et al., 2007; Bai et al., 2011). The sensing of JA-Ile by the COI1-JAZ co-receptor in the plant body causes stress reactions (Saito et al., 2021). JAZ1-7, 9-12 mutations in *Arabidopsis* improve JA responses while suppressing COI1 phenotypes in flowering time, rosette expansion, and defense (Liu et al., 2021). According to the results of protein-protein interactions, we speculate that some TIFY family members might interact with other genes, such as MYC2/MYC3 and COI1 and NINJA, for their functions, especially against different stresses. However, the interactions of cassava TIFYs could be verified by yeast one-hybrid (Y1H) assays in the future.

Previous research has shown that members of the TIFY family, as unique plant TFs, play critical roles in regulating plant responses to abiotic stressors. Thus, the increased abiotic stress tolerance of transgenic plants was linked to the overexpression of the TIFY gene family. *AtTIFY10a* and *AtTIFY10b* significantly enhanced *Arabidopsis* plant tolerance to salt and alkaline stresses (Zhu et al., 2014). Overexpression of maize *JAZ14* in *Arabidopsis* may improve seedling tolerance to hormone treatments such as ABA and JA, as well as polyethylene glycol stress (Zhou et al., 2015). Under salt stress, in soybean (*Glycine max*), *GmTIFY10e* and *GmTIFY10g* transgenic plants grew faster and had longer root lengths and fresh weights than wild-type plants (Liu et al., 2022). Similarly, virtually all TIFY genes in rice are susceptible to a variety of abiotic stresses, and *OsTIFY11a* overexpression resulted in a significant increase in tolerance to salt and dehydration stress (Ye et al., 2009). The current study found that multiple stress treatments (osmotic, salt, and cadmium) change the expression of the majority of TIFY genes in cassava, showing that cassava TIFY genes play important roles in the response to abiotic stress (**Figure 8**; **Figure 9**; **Figure 10**).

Various plant hormones, such as ABA, SA, and JA are involved in stress responses by activating the transcription of defense-related genes. In response to hormone treatments, cassava *JAZ* genes were highly regulated by JA and ABA, which is consistent with previous study of *Brassica rapa* JAZ subfamily genes (Saha et al., 2016). There is abundant evidence that JA treatment and environmental cues rapidly trigger *JAZ* gene expression, which may be responsible for moderating the JA response (Chini et al., 2007; Thines et al., 2007; Katsir et al., 2008). In this study, we found that almost cassava *MeJAZ* genes were induced and showed relative high expession compared to the control at earlier stages of JA treatment (1 and 6 h). It was hypothesized that the cassava TIFY gene family may contribute to resistance *via* the JA signaling system. ABA is important in a plant’s ability to adapt to harsh environmental circumstances (Umezawa et al., 2010). In rice, for example, ABA was found to stimulate four of the twenty TIFY genes (Ye et al., 2009). Six of the 11 abiotic stress-responsive TIFY genes studied in grape were shown to be controlled by ABA (Zhang et al., 2012). Some stress-responsive TIFY genes, such as *MeJAZ1*, *MeJAZ13*, and *MeJAZ14*, were obviously stimulated by exogenous ABA treatment in this study. These genes are syntenic paralogs of *Arabidopsis* JAZ12, which interact with MYC2 (**Figure 5**). In *Arabidopsis*, JAZ1 and JAZ3 interact with MYC2, which also acts as a transcriptional activator in ABA-inducible gene expression in plants under drought stress (Abe et al., 2003; Chini et al., 2007). Future research will need to determine whether these cassava TIFYs operate in abiotic stress response *via* interaction of the JA and ABA signaling pathways.

## Conclusion

Cassava genome-wide analysis revealed 28 TIFY family genes, including 7 *ZML*, 3 *PPD*, 16 *JAZ*, and 2 *TIFY* genes. The phylogenetic study found that the newly identified cassava *JAZ*, *PPD*, and *ZML* genes could be divided into divided into three primary clades, and JAZ were the largest group. Members of the same group having similar gene structures and motif compositions. Collinearity analysis indicated that segmental duplication events were important in the evolution of cassava *JAZ*, *PPD*, and *ZML* genes, and that all duplicated pairs had been influenced by severe purifying selection. The presence of key *cis*-regulatory elements in the promoter region of *JAZ*, *PPD*, and *ZML* genes suggests that the TIFY gene family, a collection of plant-specific TFs, is activated by a range of stimuli. According to expression profile analyses, almost all cassava *JAZ*, *PPD*, and *ZML* genes were susceptible to at least one abiotic stress and hormone treatments. In addition, we also identified several cassava *JAZ* genes, such as *MeJAZ1*, *MeJAZ13*, and *MeJAZ14*, that may potentially be involved in tolerance to environmental stresses. Our findings may be beneficial in developing a framework for future functional characterization of TIFY family in cassava.

## Data availability statement

The original contributions presented in the study are included in the article/**Supplementary Materials**. Further inquiries can be directed to the corresponding author/s.

## Author contributions

KL, YC and RZ conceived the study and worked on the approval of the manuscript. LZ, QW, and KL performed the experiments and wrote the first draft. KL, YC, RZ and XZ revised the manuscript. LZ, QW, HW, CG and XN contributed to data analysis and managed reagents. All authors contributed to the article and approved the submitted version.

## Funding

This work was supported by Natural Science Foundation of Hainan Province (2019RC112), Natural Science Foundation of China (32160324), and the China Agriculture Research System (CARS-11-hncyh).

## Conflict of interest

The authors declare that the research was conducted in the absence of any commercial or financial relationships that could be construed as a potential conflict of interest.

## Publisher’s note

All claims expressed in this article are solely those of the authors and do not necessarily represent those of their affiliated organizations, or those of the publisher, the editors and the reviewers. Any product that may be evaluated in this article, or claim that may be made by its manufacturer, is not guaranteed or endorsed by the publisher.
