# Supplementary figures and images for: Genome-wide identification and expression of TIFY family in cassava (Manihot esculenta Crantz)
Source: Front Plant Sci. 2022 Oct 5;13:1017840. doi: 10.3389/fpls.2022.1017840 (PMC9581314; doi:10.3389/fpls.2022.1017840)

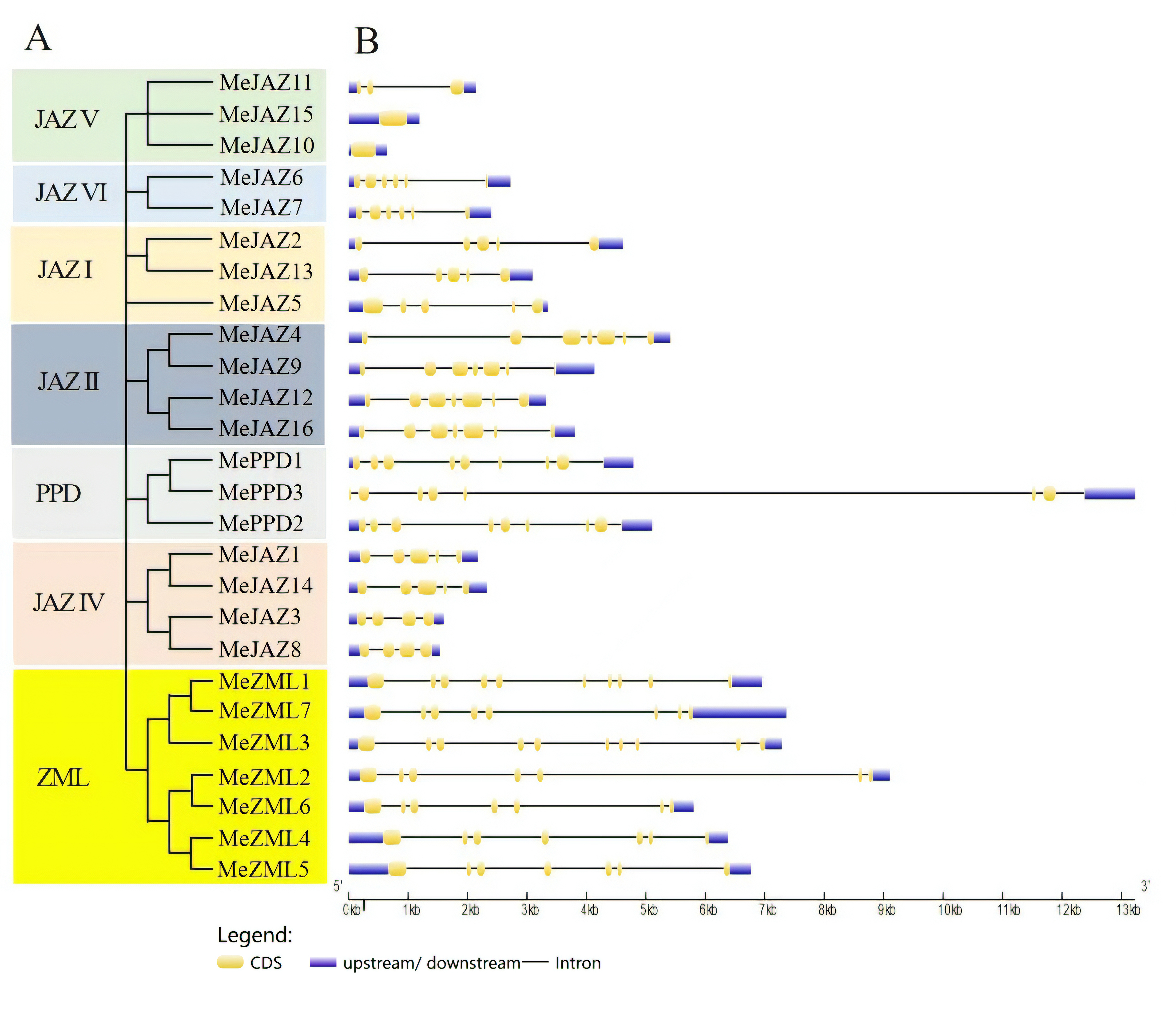

Supplement: Supplementary file 1 [file Image_1.jpeg]

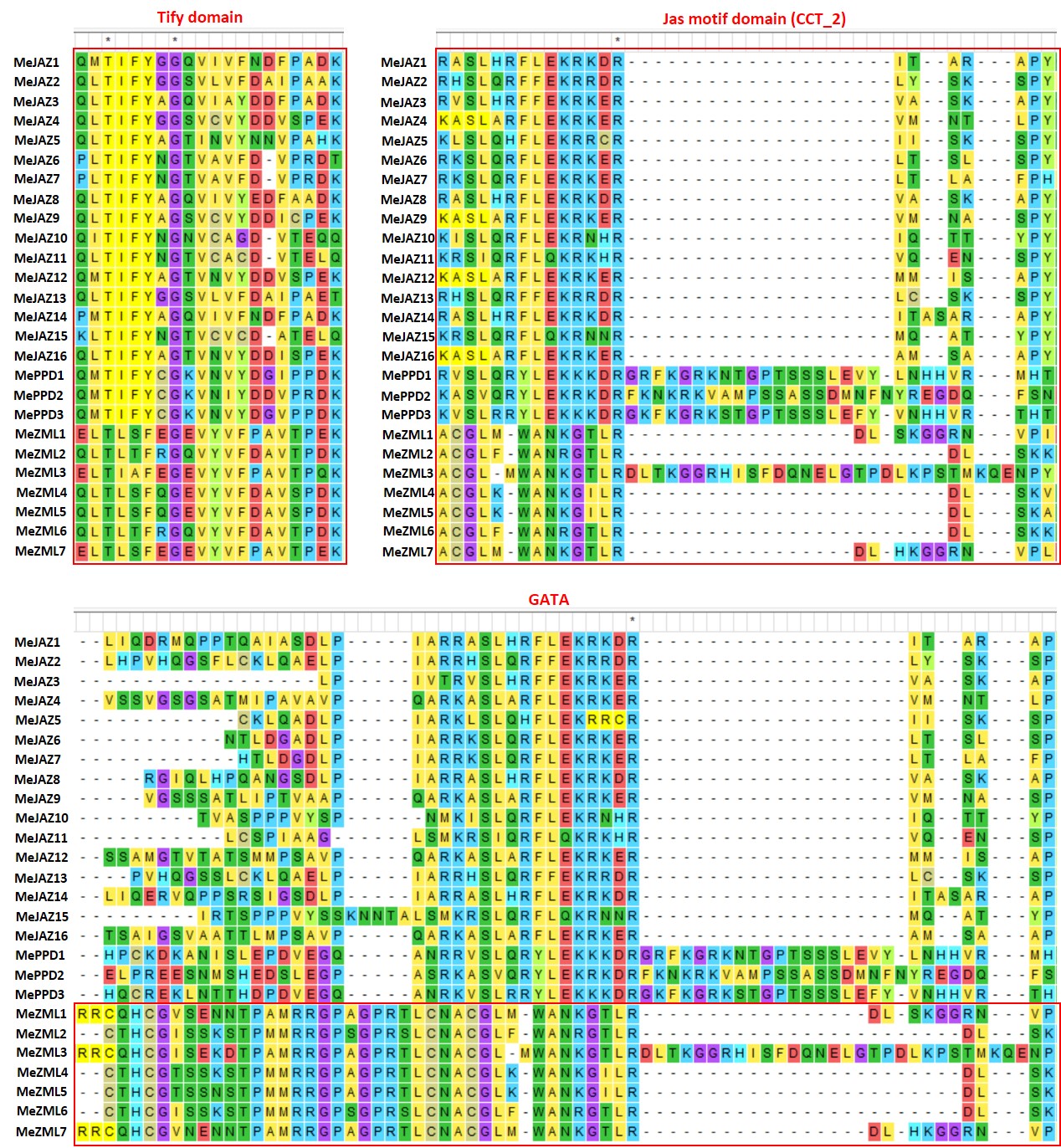

Supplement: Supplementary file 2 [file Image_2.jpeg]

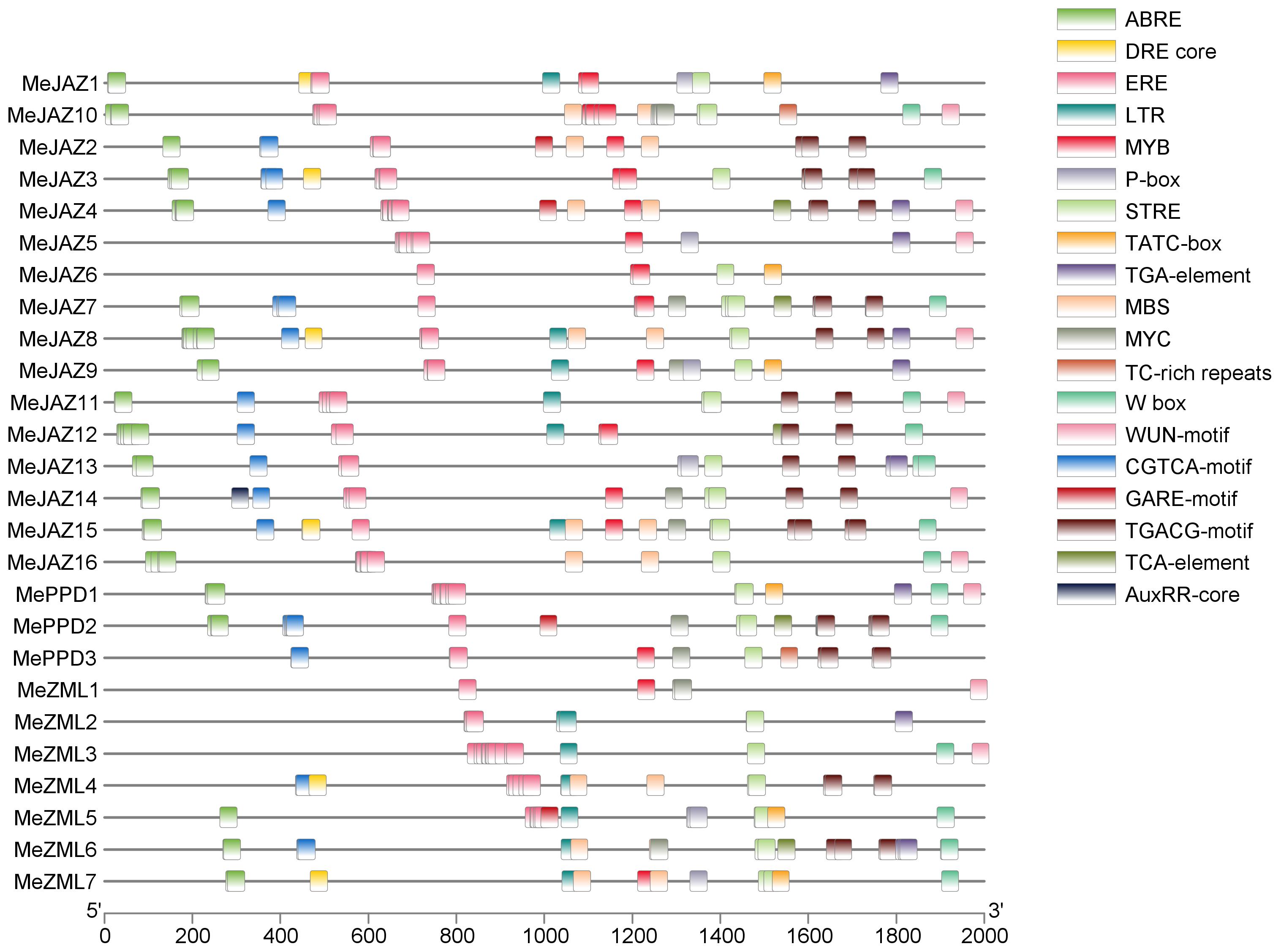

Supplement: Supplementary file 3 [file Image_3.jpeg]

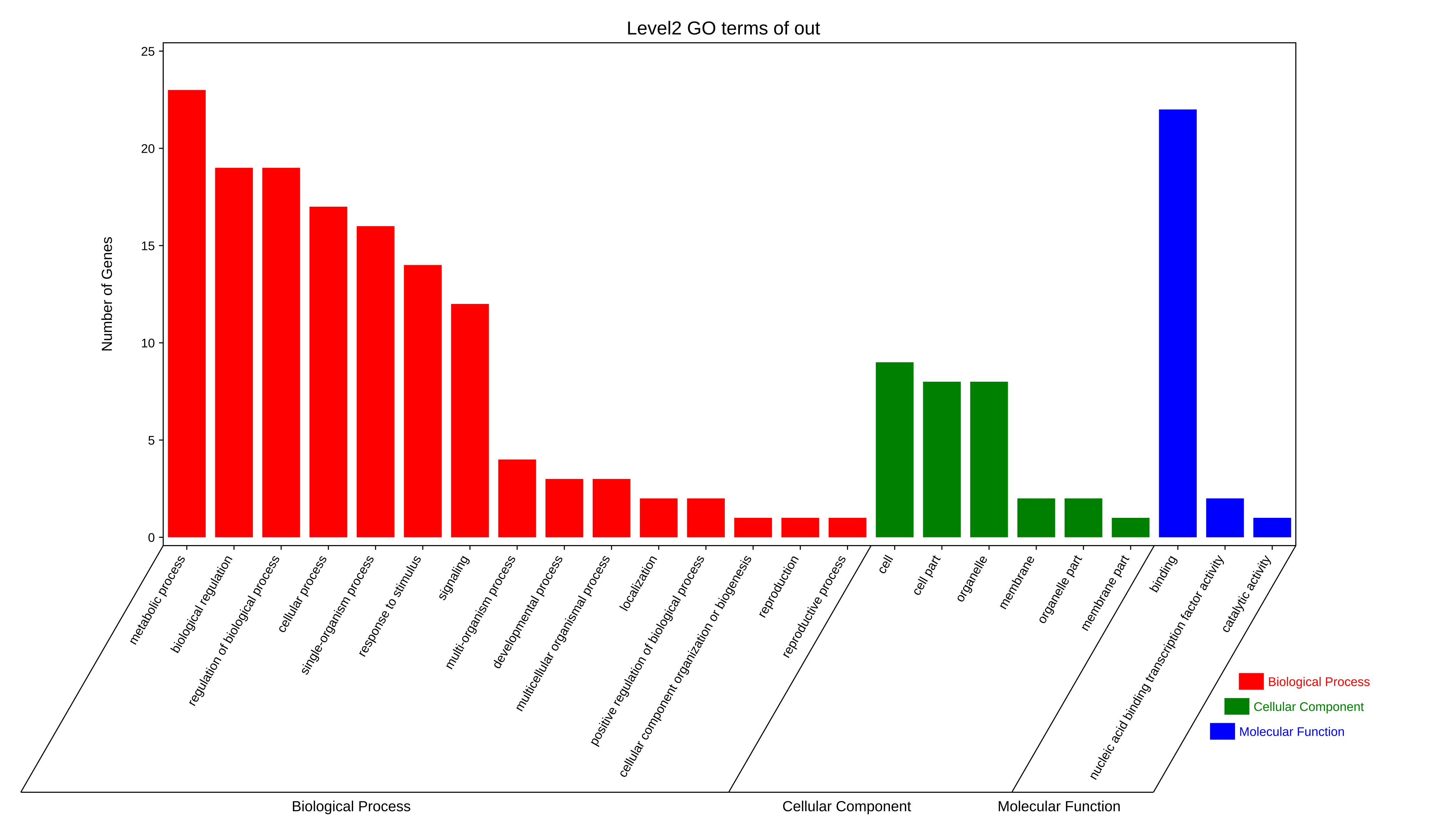

Supplement: Supplementary file 4 [file Image_4.tif]

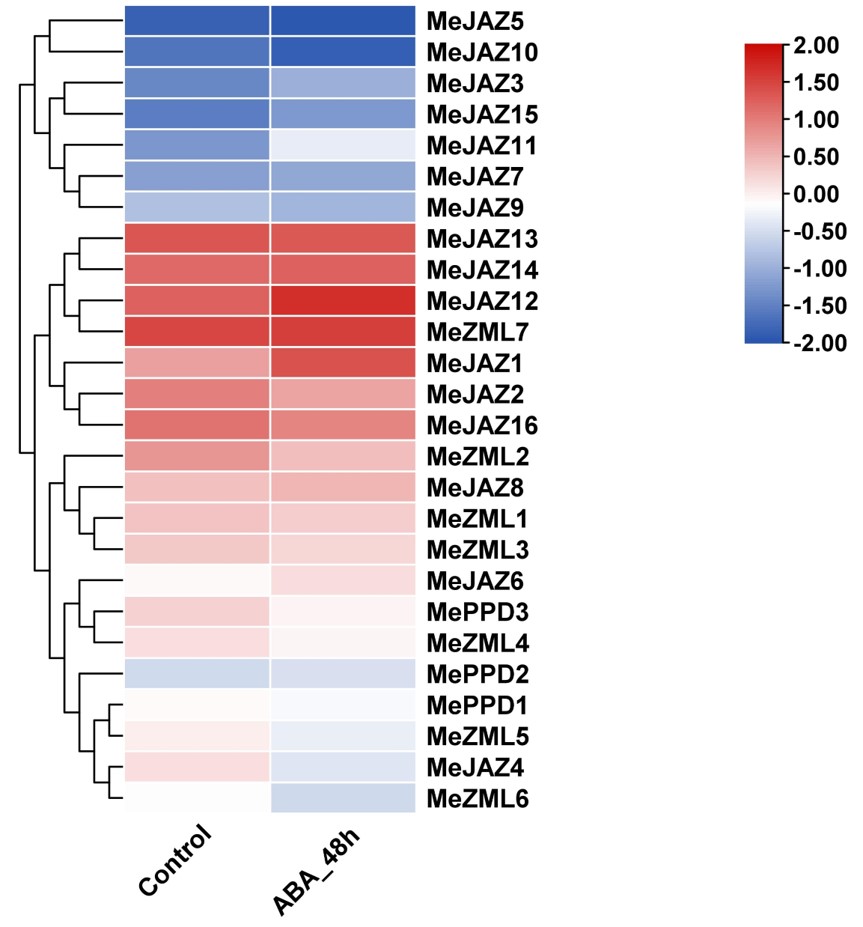

Supplement: Supplementary file 5 [file Image_5.jpeg]
